# The OsCBL8–OsCIPK17 Module Regulates Seedling Growth and Confers Resistance to Heat and Drought in Rice

**DOI:** 10.3390/ijms232012451

**Published:** 2022-10-18

**Authors:** Cong Gao, Shuai Lu, Rong Zhou, Zihui Wang, Yi Li, Hui Fang, Baohua Wang, Moxian Chen, Yunying Cao

**Affiliations:** 1 College of Life Sciences, Nantong University, Nantong 226007, China; 2 National Key Laboratory of Plant Molecular Genetics, CAS Center for Excellence in Molecular Plant Sciences, Institute of Plant Physiology & Ecology, Chinese Academy of Sciences, Shanghai 200032, China; 3 State Key Laboratory of Crop Biology, College of Life Science, Shandong Agricultural University, Taian 271000, China

**Keywords:** *Oryza sativa*, CBL, CIPK, growth, high temperature, drought

## Abstract

The calcium signaling pathway is critical for plant growth, development, and response to external stimuli. The CBL–CIPK pathway has been well characterized as a calcium-signaling pathway. However, in most reports, only a single function for this module has been described. Here, we examined multiple functions of this module. CIPK showed a similar distribution to that of CBL, and OsCBL and OsCIPK families were retained after experiencing whole genome duplication events through the phylogenetic and synteny analysis. This study found that OsCBL8 negatively regulated rice seed germination and seedling growth by interacting with OsCIPK17 with overexpression and gene editing mutant plants as materials combining plant phenotype, physiological indicators and transcriptome sequencing. This process is likely mediated by OsPP2C77, which is a member of the ABA signaling pathway. In addition, OsCBL mediated the targeting of OsNAC77 and OsJAMYB by OsCIPK17, thus conferring resistance to high temperatures and pathogens in rice. Our work reveals a unique signaling pathway, wherein OsCBL8 interacts with OsCIPK17 and provides rice with multiple resistance while also regulating seedling growth.

## 1. Introduction

Calcium plays an essential role in maintaining the stability of cell walls, cell membranes, and membrane-binding proteins. As a second messenger, calcium is widely involved in regulating plant growth, development, and responses to external stimuli [1]. Signal transduction pathways are initiated at the cell’s perception of external signals. Different environmental stimuli are regarded as primary signals; they are recognized by specific receptors and are transmitted to intracellular second messengers, inducing a downstream cascade reaction [2]. Plants respond to physiological and biochemical changes and elicit subsequent biological effects. Based on structure and function, two categories of receptors recognize Ca^2+^ signals. One is the transitive receptor with a Ca^2+^-binding domain, which has no kinase activity and which regulates the expression of downstream genes by interacting with other proteins, such as calmodulins, calmodulin-like protein, and calcineurin B-like protein (CBL). These regulate other effector proteins to induce downstream reactions and, thus, transmit signals. The second category comprises responsive receptors that bind to Ca^2+^ and exhibit kinase activity. Their functions are mediated by binding to Ca^2+^ and inducing conformational changes in calcium-dependent protein kinases [3].

CBLs belong to the family of serine/threonine protein phosphatases and have a specific Ca^2+^-binding domain, also called the EF-hand domain [4]. In rice, each CBL protein contains four conserved EF-hand domains (Supplementary Figure S1). In addition, myristoylation and palmitoylation sites exist at the N-terminal region and FPSF motifs at the C-terminus region. The myristoylation and palmitoylation sites at the N-terminal region of the CBL protein help localize the CBL protein [5]. The serine residues in the FPSF motif are phosphorylated by the CBL-interacting protein kinase (CIPK) to enhance protein–protein interactions and activate the CBL–CIPK complex to target downstream proteins [6]. During Ca^2+^-mediated signal transduction, CBL interacts with Ca^2+^-dependent serine/threonine protein kinases, namely CIPK, to transmit signals within cells [7]. CIPKs are a class of CBL-dependent protein kinases with conserved sequences. Typical CIPK proteins contain an N-terminal kinase domain and C-terminal regulatory domain (Supplementary Figure S2). An activation loop exists in the N-terminal kinase domain between the Asp–Phe–Gly and Ala–Pro–Glu motifs [8]. Protein kinases can be activated by phosphorylation of one or more amino acid residues in the activation loop [9]. The C-terminal regulatory domain contains a NAF motif that specifically binds to CBL [10]. Under normal conditions, CIPK activity is inhibited by the intramolecular binding of the NAF motif and kinase domain. After receiving a specific signal, the NAF motif binds to CBL, resulting in the activation of CIPK. In addition, toward the C-terminus of the NAF motif exists a protein phosphatase interaction motif that binds to protein phosphatase 2C (PP2C) [11]. Studies have found that CBL proteins in plants can interact with specific CIPK proteins, CIPK proteins can also interact with specific CBL proteins, and these interactions play an essential role in response to stress as well as plant growth and development [12]. For example, the interactions of CBL3 and CBL4 with CIPK24 and CBL10 with CIPK24 in *Arabidopsis* are responsible for the response to salt stress [13]. Further, CBL10 and CIPK6 are involved in immune reactions and disease-programmed cell death in tomatoes and tobacco [14]. The formation of CBL–CIPK complexes, such as the CBL1 and CIPK1 complex, requires the presence of Ca^2+^ [15]. In contrast, other CBLs, such as CBL2 and CBL4, are Ca^2+^-independent. For example, CBL2 interacts with CIPK14 and CBL4 with CIPK24 in the absence of Ca^2+^ [16]. CBLs can also interact with CIPKs from different species. For example, the CBL of rice can interact with the CIPK of *Arabidopsis* [17] and Bermuda grass [18]. Therefore, CBLs and CIPKs are relatively conserved in the plant kingdom.

Gene replication can occur in several ways, including tandem duplication, segmental duplication, transposon element-mediated duplication, and whole-genome duplication (WGD). WGD is also known as polyploidy. Polyploidization of the genome leads to chromosome doubling and eventually the retention of many repetitive genes. These repetitive genes are essential driving forces that lead to the evolution of gene functions [19]: although rapid gene deletions follow WGD, certain genes with specific functions will eventually remain [20]. For example, after WGD in *Populus*, the enriched genes were related to vesicle and protein-targeted transport, protein degradation, RNA transcription regulation, and protein post-translational modification [21]. Additionally, transcription factors and signal transduction-related genes were significantly enriched after WGD in rice and *Arabidopsis* [22].

In this study, the phylogenetic and synteny analysis of CBL and CIPK were carried out to determine whether CBL and CIPK could be retained in WGD. OsCBL2 and OsCBL8 were selected with great differences of the promoter region and gene structure, were identified from their interacting CIPK proteins using yeast two hybrid (Y2H) and bimolecular fluorescence complementation (BiFC), and were analyzed for their spatiotemporal expression pattern. CBL8 can regulate seed germination through gene editing materials of rice. Transcriptome analysis with overexpression lines of OsCBL8 and OsCIPK17 can understand the potential mechanism underlying the regulation of seedling development. Furthermore, the downstream protein of OsCIPK17 was further screened and verified for its response to high temperature and hormones. In general, this study aims to deepen our understanding of the mechanism by which the CBL–CIPK module regulates rice growth, development, and resistance, and it provides a theoretical basis for breeding excellent varieties.

## 2. Results

### 2.1. Phylogenetic Analysis of CBL and CIPK

A variety of plants, including monocotyledons, dicotyledons, bryophytes (*Physcomitrella patens*), and ferns (*Selaginella moellendorffii*), were investigated to obtain the copy number of *CBL* and *CIPK* (Supplementary Figure S3). Four copies of *CBL* were found in bryophytes and ferns. A balanced distribution of *CBL* in monocotyledons and dicotyledons was observed, with no more than 10 gene copies. In contrast, the number of *CIPK* copies varied considerably, being as high as four times the lowest number, suggesting that *CIPK* amplification occurred earlier than *CBL* during evolution. The number of *CIPK* copies was consistent, fluctuating at approximately 30. Among all investigated plants, there were 222 copies of CBL and 711 copies of CIPK.

The monocotyledon model plants rice and maize and the dicotyledon model plant *Arabidopsis* were selected to construct a phylogenetic tree using the maximum-likelihood method (Supplementary Figure S4) and Bayesian inference (Supplementary Figure S5) to ensure a reliable topological structure. The CBLs from the three plants did not cluster according to species and were scattered. The CBLs of *Arabidopsis* were closely clustered, whereas the CBLs of rice and maize were closely clustered and the clusters were interspersed, although with a scattered distribution. This suggests that the differentiation of CBL likely occurred after the divergence of monocotyledons and dicotyledons. Therefore, CBL differentiation likely occurred before the divergence of rice and maize. The phylogenetic tree for *CIPK* showed a similar distribution to that of CBL. However, whereas certain CIPKs in *Arabidopsis* were interspersed with those of rice and maize in the phylogenetic tree, this finding was not reflected in the CBL phylogenetic trees. This observation further supports the hypothesis that CIPK amplification occurred earlier than that of CBL.

### 2.2. Synteny Analysis of CBL and CIPK Families in Rice

Synteny analysis was conducted to ascertain the replication history of CBL and CIPK in rice. First, we selected two monocotyledons (*Zea mays, Setaria viridis*) and two dicotyledons (*Arabidopsis thaliana*, *Glycine max*) and constructed their syntenic relationship with rice. Supplementary Figure S6 depicts *CBL* in red, *CIPK* in blue, and other genes in gray. This demonstrates that dicotyledons have fewer *CBL* homologous genes than monocotyledons, suggesting greater differentiation of *CBL* within monocotyledons than between monocotyledons and dicotyledons. We did not find homologous genes for *CBL* in *Arabidopsis*; thus, *CBL* in rice and *Arabidopsis* has likely originated from two ancestral genes. Except for greater synteny, the results for *CIPK* were similar to those for *CBL*. Early differentiation of *CIPK* and a greater number of copies increased the syntenic relationship.

Synteny between *CBL* and *CIPK* in rice revealed 23 homologous genes in *OsCBL* and *OsCIPK* (Supplementary Figure S7). These included two pairs of tandem duplications, one pair each of *CBL* and *CIPK*. The remaining 21 pairs were fragment replications, including one pair of *CBL* and 20 pairs of *CIPK*. This is consistent with previous results showing that members of gene families are mainly produced by fragment replication [23]. We mapped the dot plot of genome-wide synteny in rice to distinguish between fragment replication and WGD (Supplementary Figure S8) and combined it with the whole genome Ks value to determine the gene pairs that experienced WGD (Supplementary Figure S9A). The results showed that four pairs of *CIPK* experienced WGD. We also estimated the time of the WGD event based on the Ks value [24]. *OsCIPK1–OsCIPK17* underwent WGD approximately 54 Mya. *OsCIPK4–OsCIPK7*, *OsCIPK5–OsCIPK20*, and *OsCIPK11–OsCIPK28* experienced WGDs approximately 97, 100, and 127 Mya, respectively. This is consistent with the WGDs of approximately 60 and 120 Mya reported in previous studies [25]. The Ka/Ks values for all homologous gene pairs (Supplementary Figure S9B) were less than 1, suggesting that *OsCBL* and *OsCIPK* were strongly purified or stabilized during evolution.

### 2.3. Specific Interaction and Spatiotemporal Expression Pattern Analysis of Sectional CBL and CIPK

**Figure 1:**
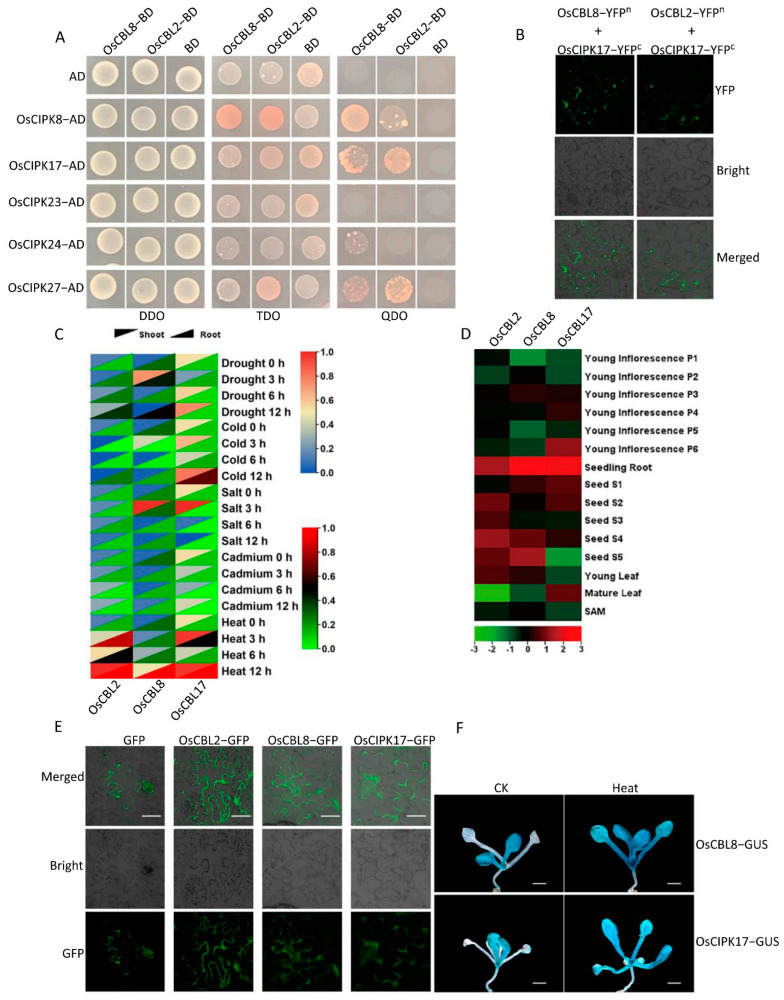
OsCBL and OsCIPK have specific interactions and different spatiotemporal expression patterns. (**A**) The interaction between OsCBL2, OsCBL8 and part of OsCIPK was confirmed by Y2H analysis. (**B**) The interaction between OsCBL2, OsCBL8 and OsCIPK17 was confirmed by BIFC analysis. (**C**) The heat maps of gene expression of *OsCBL2*, *OsCBL8* and *OsCIPK17* under different stress. (**D**) The heat maps of gene expression of *OsCBL2*, *OsCBL8* and *OsCIPK17* in different parts. (**E**) Subcellular localization of OsCBL2, OsCBL8 and OsCIPK17. Bar = 100 μm. (**F**) Gus tissue staining of Arabidopsis transformed with the promoter regions of *OsCBL8* and *OsCIPK17* under high temperature stress. The scale is 100 μm. CK, control group, without high temperature treatment; HT, experimental group, with high temperature treatment at 30 °C for one day.

We investigated the promoter region and gene structure (Supplementary Figure S10) of OsCBL and selected OsCBL2 and OsCBL8, which showed great differences, for further study (Figure 1A). Using Y2H analysis, we found that OsCBL8 interacted with OsCIPK8, OsCIPK17, OsCIPK24, and OsCIPK27. OsCBL2 interacted with OsCIPK8, OsCIPK17, and OsCIPK27. In addition, neither OsCBL8 nor OsCBL2 interacted with OsCIPK23. Although OsCBL8 and OsCBL2 interacted with the same OsCIPKs, the intensity of their interactions was obviously different. The interaction of OsCBL2 and OsCBL8 with OsCIPK17 was also detected using BiFC (Figure 1B, Supplementary Figure S11). In addition, we summarized specific interactions between CBL and CIPK, based on previous studies [17]. CBL and CIPK in *Arabidopsis* (Supplementary Figure S12A) and pumpkin (Supplementary Figure S12C) showed specific interactions similar to those in rice. Thus, CBL interacts with CIPK, although they are derived from different species (Supplementary Figure S12B).

Subsequently, we investigated the expression patterns of *OsCBL2*, *OsCBL8*, and *OsCIPK17* in the roots and shoots of rice seedlings under different stress conditions (Figure 1C). The results showed that *OsCBL2* and *OsCIPK17* had similar expression patterns in response to drought in shoots, whereas *OsCBL8* and *OsCIPK17* had similar expression patterns in roots. Under cold stress, *OsCBL2* and *OsCBL8* had different expression patterns than that of *OsCIPK17*. However, *OsCBL2* and *OsCBL8* exhibited identical root expression patterns. This suggests that *OsCBL2* and *OsCBL8* may participate in the regulation of cold stress through interactions with other OsCIPKs. *OsCBL8* and *OsCIPK17* showed similar expression patterns in shoots and roots under cadmium stress. This phenomenon also occurred under heat stress. *OsCBL8* and *OsCIPK17* showed similar expression patterns in young inflorescences at different developmental stages (Figure 1D). In different developmental stages of seeds, *OsCBL2* and *OsCBL8* had similar expression patterns but did not show similar expression patterns to OsCIPK17. A similar trend was observed for leaves. Finally, we performed subcellular localization experiments (Figure 1E). The results showed that OsCBL2 and OsCBL8 displayed strong signals in the membrane. OsCIPK17 had a signal in the cytoplasm. The BiFC results provided further confirmation, indicating that OsCIPK17 was an apparent interaction signal with OsCBL2 and OsCBL8. These results suggest that cells receiving different calcium signals recruit OsCIPK17 through OsCBL2 or OsCBL8 on the cell membrane and subsequently phosphorylate the target gene to complete the regulatory process.

Finally, we performed GUS tissue staining of *Arabidopsis thaliana* transformed with the promoter regions of *OsCBL8* genes (Figure 1F and Supplementary Figure S13). In *Arabidopsis thaliana* transformed with the *OsCBL8* promoter region, blue staining was not detected in the seed coat or embryo, indicating that *oscbl8* was not induced in these two parts. In the one-week-old seedlings, the aboveground part was stained blue, indicating that *OsCBL8* was strongly induced in the aboveground part. In one-month-old plants, there was no blue signal in the stem, whereas the signal in stem leaves formed large blue spots. This showed that *OsCBL8* was not induced in the stem, but expression signals were induced in stem leaves. Flowers and pods showed similar expression patterns in one-month-old plants. As shown in Supplementary Figure S12F–I, as development progressed, staining in the tissue gradually faded. As shown in Supplementary Figure S7F, the flower in the middle was stained dark blue and had no bud; the flower on the left was stained light blue and had just flowered. In the flower on the right, in full bloom, the blue signal could be barely detected. The pod also showed a similar pattern. As shown in Supplementary Figure S12G–I, pods in progressively late developmental stages showed gradually lighter blue staining. The GUS tissue staining results in transgenic *Arabidopsis thaliana* with the *OsCBL8* promoter region were similar to those in the transgenic *Arabidopsis* with the *OsCIPK17* promoter region, as reported in previous studies [26]. We also stained Gus tissue of 10-day-old *Arabidopsis thaliana* transformed with *OsCBL8* and *OsCIPK17* promoter regions after high temperature treatment. As shown in Figure 1F, the blue staining of both *Arabidopsis thaliana* transformed with *OsCBL8* and *OsCIPK17* promoter regions after high temperature treatment is deeper than that of the control group. This indicates that both *OsCBL8* and *OsCIPK17* can be strongly induced by high temperature.

### 2.4. OsCBL8 Regulates Seed Germination Rate and Interacts with OsCIPK17 to Regulate Seedling Growth after Germination 

**Figure 2:**
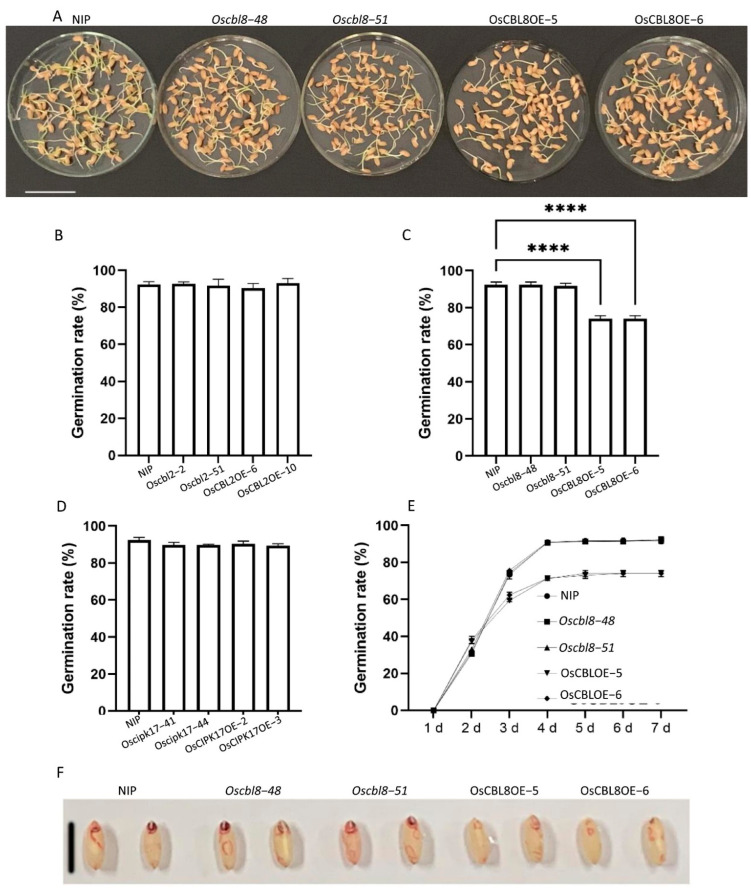
OsCBL8 can negatively regulate rice seed germination rather than OsCBL2 and OsCIPK17. (**A**) Germination phenotype of *OsCBL8* knockout and overexpression lines on seed germination of rice. Each petri dish contained 100 rice seeds. Photographs of 7-day-old plants are shown. Bar = 4 cm. (**B**) Seed germination rate of *OsCBL2* knockout and overexpression lines. (**C**) Seed germination rate of *OsCBL8* knockout and overexpression lines. (**D**) Seed germination rate of *OsCIPK17* knockout and overexpression lines. (**E**) Changes in seed germination rate of knockout and overexpression lines of *OsCBL8* in 7 days. Data of (**B**–**E**) are mean ± SD. from three biological replicates. Statistical analysis was performed using Šídák’s multiple comparisons test and Dunnett’s multiple comparisons test; **** *p* < 0.0001. (**F**) The vigor of seeds was demonstrated by TTC (2, 3, 5-triphenyltetrazole chloride) staining. After the seeds were immersed in TTC staining, they were cultured at 33 °C for 2 h and photographed for observation. The redder the seed color, the higher the seed vigor. Bar = 6 mm.

**Figure 3:**
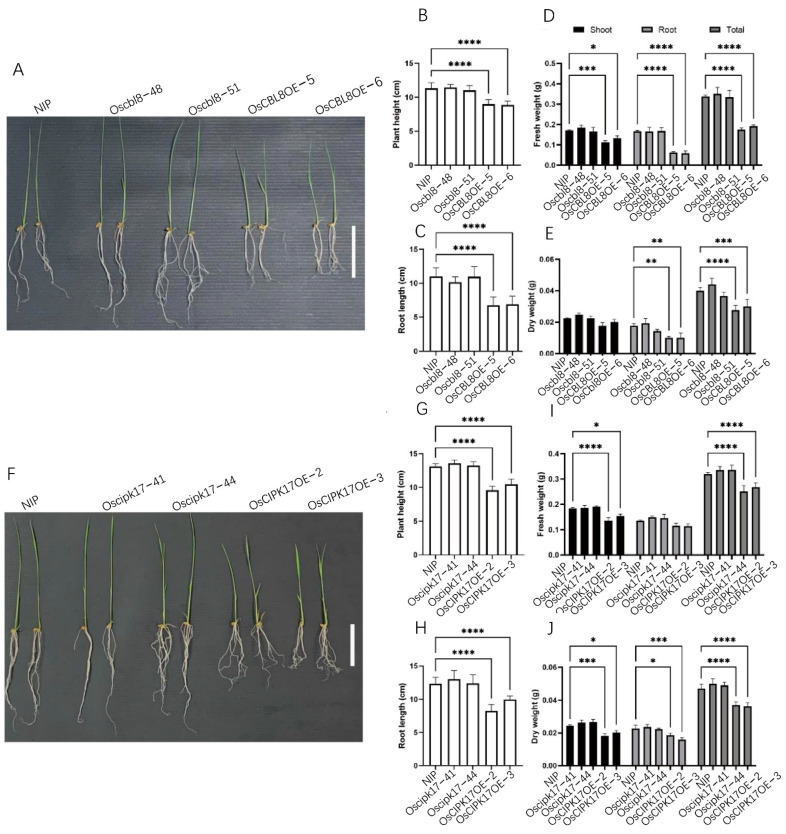
OsCBL8 and OsCIPK17 can regulate seedling growth. (**A**,**F**) Morphological phenotypes of *OsCBL8* and *OsCIPK17* knockout and overexpression lines on rice seedling growth. Photographs of 14-day-old plants are shown. Bar = 2 cm. (**B**,**C**) Plant height and root length of *OsCBL8* knockout and overexpression lines. Data are mean ± SD from 20 biological replicates for *OsCBL8.* (**D**,**E**) Fresh and dry weight of knockout and overexpression lines of *OsCBL8* were measured. (**G**,**H**) Plant height and root length of *OsCIPK17* knockout and overexpression lines. Data are mean ± SD from 12 biological replicates for *OsCIPK17*. Statistical analysis was performed using Šídák’s multiple comparisons test. (**I**,**J**) Fresh and dry weight of knockout and overexpression lines of *OsCIPK17* were measured. Data are mean ± SD from three biological replicates. Statistical analysis was performed using Dunnett’s multiple comparisons test; * *p* < 0.05, ** *p* < 0.01, *** *p* < 0.001, **** *p* < 0.0001.

To study the functions of OsCBL and OsCIPK with NIP as the parent, we constructed mutant and overexpression lines of *OsCBL2*, *OsCBL8*, and *OsCIPK17*. We found that *OsCBL8* regulated seed germination (Figure 2A). The mutant lines *oscbl8-48* and *oscbl8-51* of *OsCBL8* showed germination rates similar to those of NIP (Figure 2C). Moreover, the germination rates of the overexpression lines OsCBL8OE-5 and OsCBL8OE-6 were significantly decreased (Figure 2C). TTC staining results showed that NIP and the mutant lines showed deeper staining and were brighter red, especially in the embryo (Figure 2F). This staining pattern suggests that seed vigor, especially embryo vigor, of the overexpression lines was lower than that of the mutant lines and NIP. However, the germination rate of the mutant plants and overexpression lines of *OsCBL2* or *OsCIPK17* were not significantly different from those of NIP (Figure 2B,D). We also calculated the germination rate of the *OsCBL8* transgenic plants within seven days and found that the rates of the transgenic plants and parents began to stabilize on the fourth day (Figure 2E). However, the germination rate of the overexpression lines was higher than that of the mutant plants on the second day (Figure 2E). We also found that the *OsCBL8* and *OsCIPK17* overexpression lines inhibited seedling growth (Figure 3A,F). The plant height and root length of *OsCBL8* and *OsCIPK17* overexpression lines were significantly reduced compared with those of their parents (Figure 3B,C,G,H). In contrast, the mutant plants were similar to or larger than their parents, but the differences were not statistically significant (Figure 3B,C,G,H). We also measured the weight of the transgenic plants. The results showed that overexpression of *OsCBL8* significantly reduced the fresh weight of roots and shoots (Figure 3D). Overexpression of *OsCIPK17* also considerably reduced the fresh weight of roots but did not significantly reduce the dry weight of shoots (Figure 3I,J). However, overexpression of *OsCBL8* and *OsCIPK17* significantly reduced the dry weight of whole seedlings (Figure 3E,J). The significant reduction in fresh and dry weight corresponded with the increased water loss of the overexpression lines. 

### 2.5. Transcriptome Analysis of OsCBL8 and OsCIPK17 Overexpression Lines 

**Figure 4:**
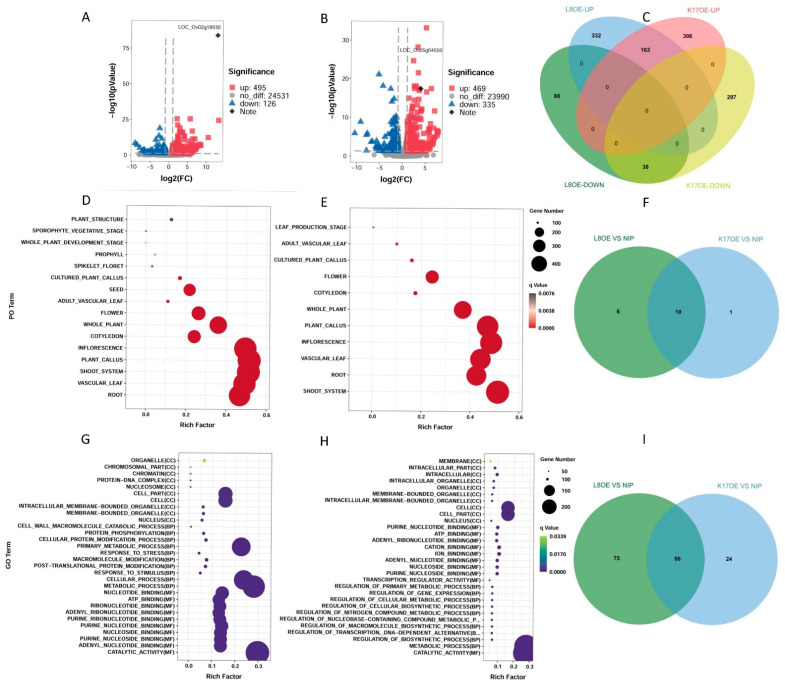
Transcriptome analysis of OsCBL8 and OsCIPK17 overexpressing lines. (**A**) Volcano map showing the changes in gene expression in overexpression lines of OsCBL8 and NIP. (**B**) Volcano map showing the changes in gene expression in overexpression lines of OsCIPK17and NIP. OsCBL8 and OsCIPK17 are marked with a diamond. (**C**) Venn diagram showing the intersection of up- and downregulated differentially expressed gene (DEG) sets. (**D**) Plant Ontology (PO) enrichment analysis of OsCBL8 overexpression lines. (**E**) Plant Ontology (PO) enrichment analysis of OsCIPK17 overexpression lines. (**F**) The Venn diagram is used to show the common terms produced by different PO enrichment analysis. (**G**) Gene Ontology (GO) enrichment analysis of OsCBL8 overexpression lines. (**H**) Gene Ontology (GO) enrichment analysis of OsCIPK17 overexpression lines. Only the top 10 terms from MF, BP and CC are shown. (**I**) The Venn diagram is used to show the common terms produced by different GO enrichment analyses.

To understand the potential mechanism underlying the regulation of seedling development and the function of *OsCBL8* and *OsCIPK17*, we selected 14-day-old seedlings from the overexpression lines *L8OE-5*, *K17OE-3*, and the parent NIP for complete transcriptome sequencing. A total of 25,152 genes were detected in the *OsCBL8* overexpression lines (Figure 4A). Of these, 495 genes were significantly upregulated, and 126 genes were significantly downregulated (Figure 4A). In the *OsCIPK17* overexpression line, 24,794 genes were identified, of which 469 were significantly upregulated and 335 were downregulated (Figure 4B). As shown in Figure 4C, 163 genes appeared in both upregulated genes sets and 38 genes in the downregulated gene sets. However, the set of upregulated genes did not intersect with the set of downregulated genes. This indicated that the overexpression of *OsCBL8* and *OsCIPK17* resulted in a highly consistent synergy in the regulation of gene expression.

We performed Plant Ontology (PO) enrichment analysis using Gene Set Enrichment Analysis (GSEA), which is also sensitive to non-differentially expressed genes. The overexpressed *OsCBL8* or *OsCIPK17* was significantly enriched in roots and shoots (Supplementary Figure S14). Subsequently, we performed a PO enrichment analysis of the differentially expressed genes (DEGs) of overexpressed *OsCBL8* or *OsCIPK17* plants (Figure 4D,E). The results showed that the DEGs were enriched in 16 and 11 terms, respectively, and 10 intersections were generated after the overexpression of *OsCBL8* and *OsCIPK17* (Figure 4F). Both were enriched in roots, shoots, leaves, flowers, and calli. The only terms enriched by overexpression of *OsCBL8* included seeds. This is consistent with a previous observation that *OsCBL8* regulates seed germination, whereas *OsCIPK17* does not.

Furthermore, we performed Gene Ontology (GO) enrichment analysis (Figure 4G,H). The two groups of DEGs were enriched in 161 and 110 terms, respectively, and produced 86 intersections (Figure 4I). For molecular function (MF), biological process (BP), and cellular component (CC), we selected the top 10 terms with the most significant enrichment degree scores (Figure 4G,H). In MF, after the overexpression of *OsCBL8*, DEGs were enriched in 63 terms, including substance binding, enzyme activity, and transporter activity. The substance binding terms included nucleoside/nucleotide binding, ATP binding, metal ion binding, and polysaccharide binding. Terms related to enzyme activity included kinase, hydrolase, peroxidase, and ATPase activity. Transporters included sugar, carbohydrate, and ion transmembrane transporters and substrate-specific transporters. In BP, DEGs were enriched in 81 terms, mainly related to metabolism, response to stimulation, reproductive development, and protein modification. For instance, metabolism included primary, chitin, polysaccharide, and protein metabolism. Responses to oxidative stress, chemical stimuli, and biological stimuli were included in the response to stimuli. Reproductive development included terms such as pollen–pistil interaction, pollination, and reproductive processes. Protein modification included cellular protein and post-translational modification. In CC, DEGs were enriched in 17 terms, including the nucleus, membrane, and organelles that bind to membranes. Overexpression of *OsCIPK17* resulted in the enrichment of DEGs, which was consistent with that of *OsCBL8*. However, there were some differences between the groups. For example, most of the terms enriched in the overexpression line of *OsCIPK17* were related to substance synthesis. Nevertheless, the terms enriched exclusively in the overexpression line of *OsCBL8* were more complex than those of *OsCIPK17*.

Finally, we used GSEA for Kyoto Encyclopedia of Genes and Genomes (KEGG) enrichment analysis and obtained 15 and 14 highly enriched terms from the *OsCBL8* (Supplementary Figure S15) and *OsCIPK17* overexpression lines (Supplementary Figure S16), respectively. Their specific enrichment information, including enrichment score (ES), normalized enrichment score (NES), *p* value, and q value, is depicted in different tables (Supplementary Tables S2 and S3). After overexpression of *OsCBL8* and *OsCIPK17*, both were enriched in five terms: ABC transporters, ether lipid metabolism, glycerophospholipid metabolism, phagosome proteasome, and soluble N-ethylmaleimide-sensitive factor attachment protein receptors (SNARE) interactions in vesicular transport. Terms enriched only in the *OsCBL8* overexpression line were alpha-linolenic acid metabolism, diterpenoid biosynthesis, endocytosis, fatty acid metabolism, pentose phosphate pathway, peroxisome, phenylalanine, plant–pathogen interaction, and protein processing in the endoplasmic reticulum. Terms enriched only in the *OsCIPK17* overexpression line were circadian rhythm plant, glutathione metabolism, N-glycan biosynthesis, oxidative phosphorylation, phosphatidylinositol signaling system, RNA degradation, sphingolipid metabolism, and ubiquitin-mediated proteolysis. 

### 2.6. Protein–Protein Interaction Network Analysis of DEGs

**Figure 5:**
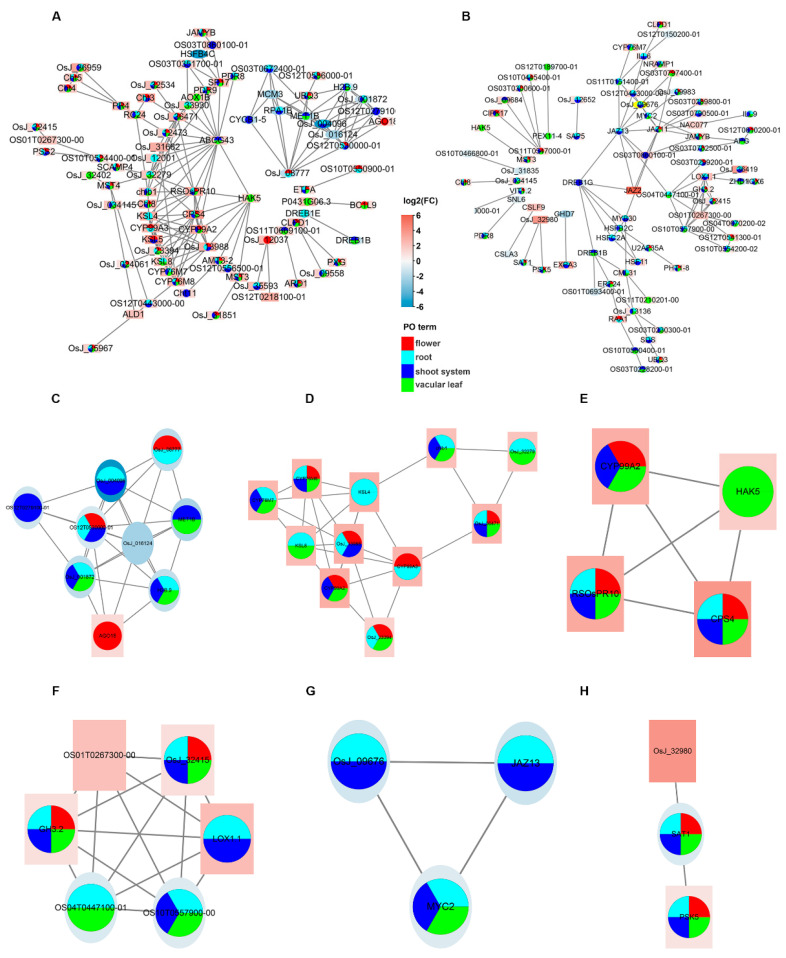
Protein–protein interaction network analysis of DEGs. (**A**) Protein–protein interaction networks generated by DEGs from *OsCBL8* and (**B**) *OsCIPK17* overexpression lines. The up- (square, red) and downregulated (round, blue) genes form two clusters, which are distinguished by different shapes and colored with log2(FC). Different PO terms are also mapped to proteins. (**C**,**D**) and (**E**) Sub-modules mined by MCODE from *OsCBL8* overexpression lines. According to the score, the top three are shown. The scores of them were 6.75, 5.2 and 4, respectively. (**F**–**H**) Sub-modules mined by MCODE from *OsCIPK17* overexpression lines. According to the score, the top three are shown. The scores of them were 6, 3 and 3, respectively.

We constructed a protein–protein interaction (PPI) network to further understand the regulatory relationships between DEGs (Figure 5A,B). At the same time, we mapped part of the results of PO enrichment onto the interaction network to predict its impact on the rice phenotype. Figure 5A shows that the upregulated and downregulated proteins clustered into two groups in plants overexpressing *OsCBL8*. In general, the interactions between upregulated proteins are more complex than those between downregulated proteins because of their large numbers. This means that the interaction between different proteins was not concentrated in the upregulated or downregulated proteins. These uniformly distributed interactions were also observed after the overexpression of *OsCIPK17* (Figure 5B). The interaction between upregulated and downregulated proteins in plants overexpressing *OsCBL8* was much weaker than those overexpressing *OsCIPK17*. In addition, some proteins showed higher degrees of interaction. For example, proteins syn-copalyl diphosphate synthases 4 (CPS4), syn-pimara-7,15-diene synthase 4 (KSL4), Jasmonate-zim-domain protein 13 (JAZ13), and OS10T0557900-00 had larger numbers of interactions. The PPI network formed by the overexpression of *OsCBL8* contained 82 annotated proteins. Among these, 54, 51, 58, and 41 proteins were enriched in roots, vascular leaves, shoot systems, and flowers, respectively. The PPI network formed by the overexpression of *OsCIPK17* contained 81 annotated proteins. Among these, 52, 50, 61, and 27 proteins were enriched in the root, vascular leaves, shoot systems, and flowers, respectively.

Further analysis of the network was conducted to screen for the important sub-modules. The top three sub-modules with the highest scores are shown in Figure 5. There were nine nodes and 27 interactions in sub-module 1 (Figure 5C) in the *OsCBL8* overexpression line, and they were enriched in the four selected PO terms. The other two sub-modules were also enriched in the four selected PO terms. According to the annotation, node AGO18 encodes a protein called Argonaute 18, which may be involved in the RNA silencing pathway. Methyltransferase 1B (MET1B) encodes DNA (cytosine-5)-methyltransferase 1b, which methylates CpG residues and maintains DNA methylation. The proteins encoded by H2B.9, OS12T0530000-01, OsJ_001872, OsJ_004096, and OsJ_016124 are histone components. The protein encoded by OS12T0279100-01 has a jmjC domain and is a histone-modified hydroxylase. OsJ_08777 is involved in the control of eukaryotic DNA replication. Notably, only AGO18 was upregulated. This suggests that sub-module 1 may regulate RNA expression by influencing DNA methylation and histone modification. There were 11 nodes and 26 interactions in module 2 (Figure 5D). Except for chitinase encoded by chib1 and a non-annotated protein (OsJ_32279), the proteins encoded by the other nine nodes were related to rice antitoxin and disease resistance. This indicates that sub-module 2 can trigger plant immunity by affecting chitin. However, all 11 nodes were upregulated, suggesting a synergistic role in regulatory processes. There were four nodes and six interactions in module 3 (Figure 5E). One (HAK5) of the four nodes encodes a high-affinity potassium transporter. The other three encode plant defense-related proteins, suggesting that the defense response of rice is also involved in the transport of potassium ions. 

There were six nodes and fifteen interactions in sub-module 1 (Figure 5F) in the *OsCIPK17* overexpression line, and they were enriched in the four selected PO terms. Four nodes were upregulated and two were downregulated. Among them, LOX1.1 and OS04T0447100-01 encode lipoxygenase, which may be involved in many aspects of plant physiology, including growth and development, insect resistance, senescence, and response to injury. GH3.2 encodes indole-3-acetic acid amino synthetase, which likely catalyzes the synthesis of indole-3-acetic acid (IAA)-amino acid conjugates, thus providing a mechanism for plants to cope with excessive auxin. OS01T0267300-00, OS10T0557900-00, and OsJ_32415 encode serine protease inhibitors, matrix metalloproteinases (MMPs), and glycogenin glucosyltransferases, respectively. The extracellular matrix (ECM) is a complex network of macromolecules surrounding cells in multicellular organisms. It is mainly composed of four kinds of substances: collagen, non-collagen, elastin, and proteoglycan-aminoglycan. ECM can support cells, retain water, and connect cells to form tissues and organs. Furthermore, it also plays an important role in cell growth, death, polarity, shape, migration, and metabolism [27]. MMP is a type of zinc and calcium-dependent protease that targets and degrades many proteins in ECM. There were three nodes and three interactions in sub-modules 2 (Figure 5G) and 3 (Figure 5H). The proteins in sub-module 2 were not enriched in flowers. In sub-module 2, JAZ13 encodes a repressor of jasmonate (JA) response. Myelocytomatosis protein 2 (*MYC2*) encodes a transcription factor involved in the transcriptional activation of the JA signaling pathway during spikelet development. OsJ_09676 encodes for an FCS such as the zinc finger (FLZ) family protein. It carries a zinc finger and acts as a PPI module. Sub-module 3 contained a downregulated node and two upregulated nodes. Phytosulfokines 5 (PSK5) promotes plant cell differentiation, organogenesis, somatic embryogenesis, and cell proliferation. OsJ_32980 encodes a pan-domain protein that participates in PPI or protein-carbohydrate interactions. Sensitive to ambient temperature 1 (SAT1) encodes a tRNA-modified enzyme that is sensitive to environmental temperature. Additionally, we analyzed the correlation between each sub-module. The results showed that almost all nodes in the sub-module were correlated (Supplementary Figure S17).

Finally, we selected hub genes from several perspectives, including maximal clique centrality (MCC) and degree. Supplementary Figure S18A shows that the screened hub genes and members in the sub-modules have many repetitions. For example, root specific rice PR10 (*RSOsPR10*) encodes for a root-specific pathogenesis-related protein. Some hub genes did not overlap with members of the submodule. For instance, ABC transporter G family member 43 (*ABCG43*) encodes a defense protein. In addition, all the sub-modules obtained from the overexpression line of *OsCIPK17* contained hub genes (Supplementary Figure S18B). Other hub genes encode stress-related proteins, such as dehydration-responsive element-binding proteins. These results indicate that the *OsCBL2*-*OsCIPK17* complex participates in the regulation of seed defense.

### 2.7. OsCIPK17 Interacts with OsPP2C77, OsJAMYB, OsNAC77, and OSDREB1H to Regulate Rice Growth and Stress Resistance

**Figure 6:**
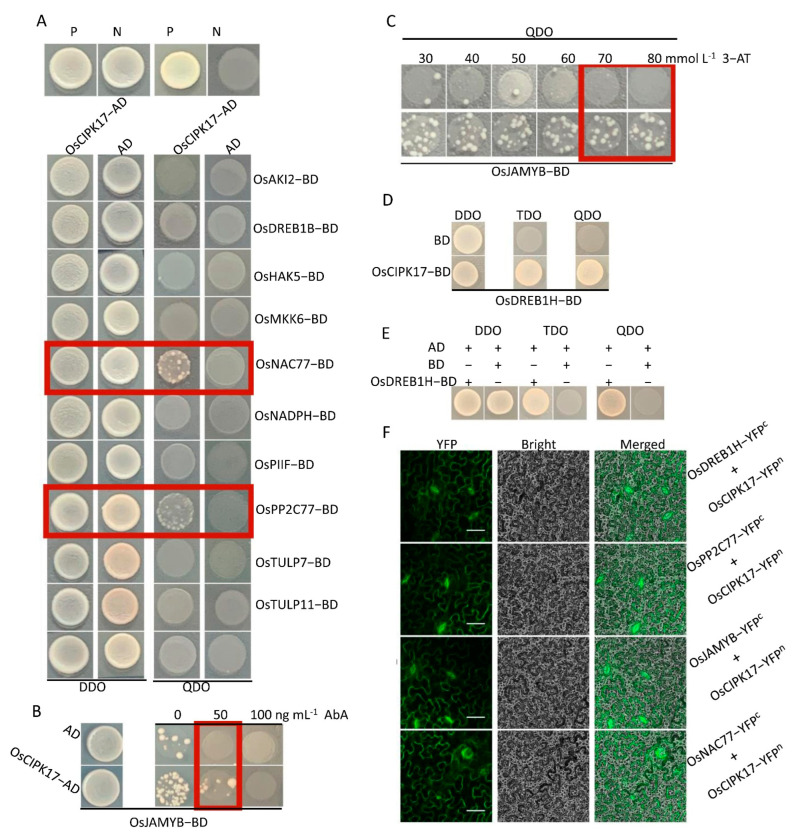
OsCIPK17 can interact with OsPP2C77, OsJAMYB, OsDREB1H and OsNAC77. (**A**) The Y2H experiment verified the target protein that may have a regulatory relationship with OsCIPK17. P: positive control; N: negative control. (**B**,**C**) Y2H experiment verified that OsCIPK17 can interact with OsJAMYB. Aureobasidin A (AbA) and 3-amino-1, 2, 4-triazole (3-AT) were added to QDO medium to inhibit self-activation. (**D**) The Y2H experiment verified that OsCIPK17 can interact with OsDREB1H. (**E**) Detection of trans-activatory activity of OsDREB1H. A plus sign indicates that the ingredient has been added, and a minus sign indicates the opposite. (**F**) BiFC experiments verified that OsCIPK17 can interact with OsNAC77, OsJAMYB, OsPP2C77 and OsDREB1H, respectively. Bar = 100 μm.

**Figure 7:**
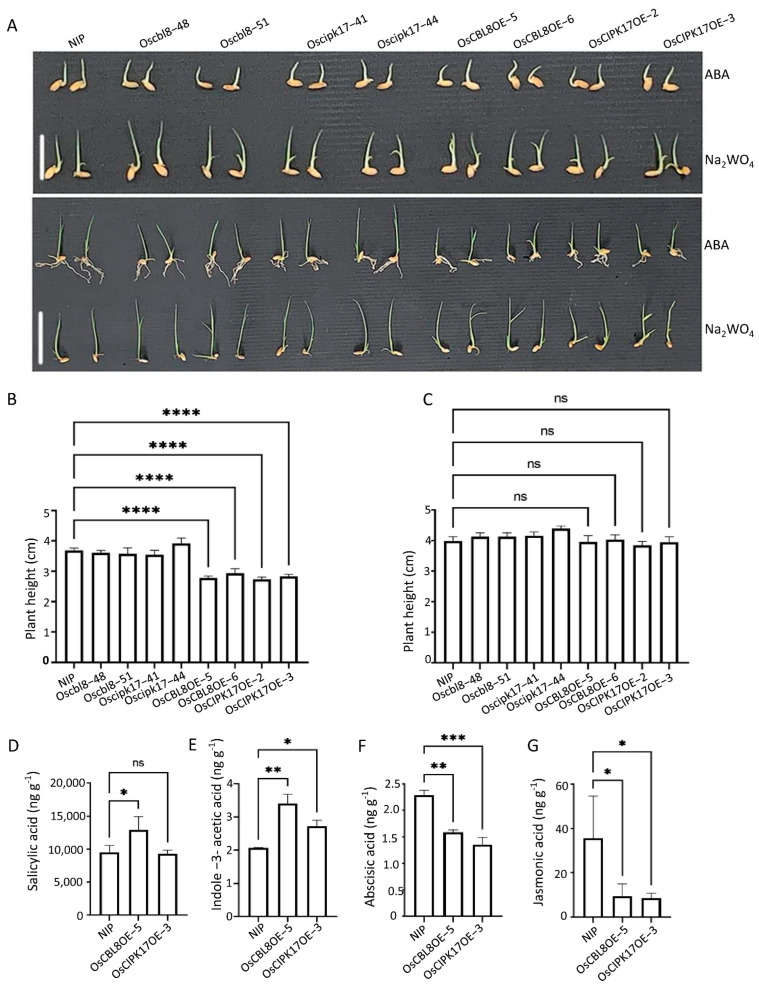
ABA participates in OsCBL8–OsCIPK17 module to regulate the growth of rice seedlings. (**A**) Phenotypes of seeds treated with 5 mg L^−1^ ABA and 100 mg L^−1^ Na_2_WO_4_ for 1 and 2 weeks, respectively. The bars are 2 and 4, respectively. (**B**,**C**) Plant height of seeds treated with 5 mg L^−1^ ABA and 100 mg L^−1^ Na_2_WO_4_ for 2 weeks. Data are mean ± SD from 10 biological replicates. Statistical analysis was performed using Dunnett’s multiple comparisons test; **** *p* < 0.0001; ns: no significance. (**D**–**G**). Determination of hormone content in leaves of 2-week-old seedlings of different lines. Data are mean ± SD from three biological replicates. Statistical analysis was performed using Dunnett’s multiple comparisons test; **** *p* < 0.0001; ns: no significance. * *p* < 0.05, ** *p* < 0.01, *** *p* < 0.001; ns: no significance.

To study the specific mechanism by which OsCBL8–OsCIPK17 regulates growth and development in rice, we predicted a regulatory relationship between some proteins and OsCIPK17, based on the results of transcriptome analysis. In the functional enrichment analysis of DEGs, we found that overexpression of the OsCBL8–OsCIPK17 module can affect the response of plants to different stresses, such as oxidative stress, chemical stimuli, and biological stimuli. At the same time, we combined some reported CIPK target genes to predict potential CIPK17-interacting proteins. As shown in Figure 6, OsJAMYB, OsNAC77, and OsPP2C77 interacted with OsCIPK17. *OsPP2C77* encodes an ABA-mediated phosphatase that interacts with CIPK [11]. This result suggests that ABA may be involved in the regulation of seedling growth and development by regulating OsCIPK17. Subsequent experiments provided further supporting evidence (Figure 7A). After a week of ABA treatment, no significant changes were observed among the seedlings of the different lines. Treatment with Na_2_WO_4_ showed the same trend. However, regardless of ABA or Na_2_WO_4_, seedling growth and development were affected after a week of treatment. After a two-week treatment, ABA-exposed seedlings showed significant differences, with the plant height of the overexpression lines being significantly lower than that of NIP (Figure 7B), as expected for seedlings grown under normal conditions without any treatment. However, seedling growth after ABA treatment was worse than that under normal conditions. After Na_2_WO_4_ treatment, no differences in plant height were observed between the overexpression lines and NIP, although growth was worse than that without treatment (Figure 7C). This shows that the inhibition of ABA metabolism can compensate for the overexpression of *OsCBL8* and *OsCIPK17* to a certain extent. Subsequently, we quantitatively detected hormones that affect plant growth and development and the defense response to verify our experimental results. Overexpression of *OsCBL8* caused an increase in salicylic acid (SA) levels; there was no significant change in lines overexpressing OsCIPK17 (Figure 7D). Indole-3-acetic acid (IAA) levels showed an upward trend in both overexpression lines (Figure 7E). In contrast, ABA and jasmonic acid (JA) levels decreased (Figure 7F,G). These results suggest that the OsCBL8–OsCIPK17 module can regulate growth and development and defense strategies by regulating IAA, ABA, and JA levels. A recent study has shown that SA is involved in plant hyperthermia and immune responses [28]. However, whether the regulation function of this module depends on SA requires further study. 

**Figure 8:**
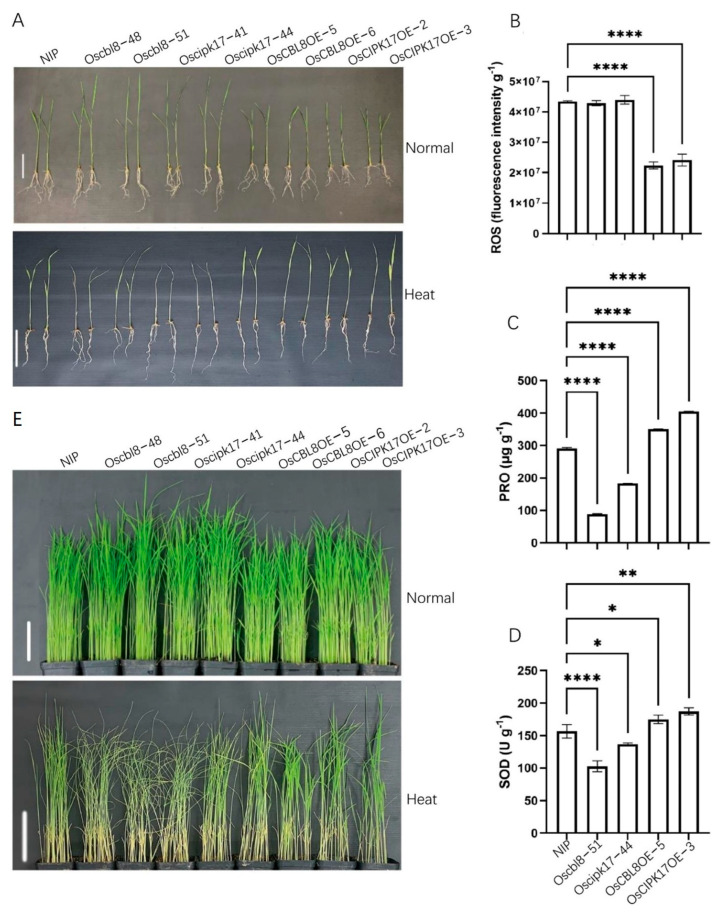
OsCBL8-OsCIPK17 can regulate the resistance to high temperature and drought. (**A**) Phenotype of 2-week-old seedlings after 9 days of high temperature treatment. Bar = 8 cm. (**B**–**D**) Measurement of indexes after high temperature stress. The contents of ROS, PRO and SOD activity were determined. ROS: reactive oxygen species; PRO: proline; SOD: superoxide dismutase. Data are mean ± SD from three biological replicates. Statistical analysis was performed using Dunnett’s multiple comparisons test; **** *p* < 0.0001, ** *p* < 0.01, * *p* < 0.05. (**E**) Phenotype of 22-day-old rice seedlings after 12 days of drought treatment. Bar = 10 cm.

In addition, we tested the response of OsCBL8–OsCIPK17 to high temperatures. Figure 8A and Supplementary Figure S19 show that the leaves of the mutants withered and curled, whereas the leaves of the overexpression plants were still in the unfolded state after high-temperature stress. The determination of physiological indices further supports this observation. After the high-temperature treatment, the overexpression lines had lower levels of reactive oxygen species (ROS) (Figure 8B) and higher proline (PRO) than parents (Figure 8C). In addition, superoxide dismutase (SOD) activity was higher in the overexpression lines than in the parent, while that of corresponding mutants was low (Figure 8D). This indicates that OsCBL8-OsCIPK17 can positively regulate seedling resistance to high temperatures. 

Additionally, we conducted experiments to simulate drought. Figure 8E shows the phenotype of the seedlings grown for 22 days under normal conditions and then not watered for 12 days. The figure shows that the leaves of the mutant lines withered and almost died, while the leaves of the overexpressed lines were normal. The leaves of NIP showed an intermediate response between the two extremes. In summary, the interaction of OsCIPK17 with OsPP2C77, OsJAMYB, and OsNAC77 was found to affect the growth of rice seedlings, regulate their response to high temperature, and confer resistance to pathogens. OsCBL8 mediates the latter function. In addition, we identified a DERB family member protein that interacted with OsCIPK17 (Figure 6D) and demonstrated its trans-activation properties (Figure 6E). However, whether this protein is involved in the response of rice to high temperatures and drought stress requires further study.

## 3. Discussion

Cellular calcium signaling regulates almost every aspect of eukaryotic physiology (Tang et al., 2020). CBL–CIPK, an important calcium-signaling module, has attracted considerable attention from botanists since it was first discovered in *Arabidopsis* [29]. We investigated the number of *CBLs* and *CIPKs* in some plants, including monocotyledons, dicotyledons, bryophytes, and ferns. The 222 copies of CBL and 711 copies of *CIPK* in these plants show significant quantitative differences among different plant species. A single pair of CBL–CIPK is usually present in green algae [30], and it appears that an increase in CBL–CIPK genes accompanied plant evolution. During evolution, higher plants showed a greater extent of adaptation than lower plants. For example, multiple CBL–CIPK modules play a role in pollen tube growth, a special developmental process unique to flowering plants [31]. The amplification of *CBL* and *CIPK* in *Arabidopsis* during evolution has been studied in detail in a recent report [32]. In this study, we demonstrated an increase in CBL–CIPK numbers in rice. Overall, evolutionary forces have determined and maintained the number of CBL–CIPK in different plants.

The interaction and expression patterns of CBL and CIPK in rice were analyzed to determine their identity and functional relationships after WGD. The results of the Y2H experiments showed that OsCBL8 interacts with OsCIPK24, whereas OsCBL2 does not. In addition, OsCBL8 and OsCBL2 interact with OsCIPK8, OsCIPK17, and OsCIPK27. However, they show varying degrees of interactions. For instance, the OsCBL8–OsCIPK8 interaction was more robust than that of OsCBL2–OsCIPK8, and the opposite was observed between OsCBL8–OsCIPK17 and OsCBL2–OSCIPK17. These results indicate that CBL and CIPK in rice have specific interactions. Even if the interaction objects between different CBLs are repeated, specificity is maintained by controlling the intensity of interaction. This specificity has also been demonstrated in *Arabidopsis* and pumpkins [32]. Specific interactions between CBL and CIPK have been observed in different species [17]. To verify this, we selected OsCIPK17, which interacts with both OsCBL8 and OsCBL2, and studied its expression patterns in detail. OsCBL8–OsCIPK17 and OsCBL2–OsCIPK17 showed various trends when exposed to different stresses. For example, *OsCBL2* and *OsCIPK17* showed similar expression patterns in shoots under drought conditions, whereas *OsCBL8* and *OsCIPK17* showed similar expression patterns in roots. *OsCBL8* and *OsCIPK17* exhibited identical expression patterns in young inflorescences at different developmental stages. In addition, the subcellular localization results showed that both OsCBL8 and OsCBL2 were expressed on the cell membrane and interacted with OsCIPK17. The results of GUS tissue staining also suggest that OsCBL8 can interact with OsCIPK17 to regulate plant growth and development and respond to high temperature stress. These results indicate that CBL and CIPK exhibit different spatial and temporal expression patterns at the gene, protein, and tissue levels. The specific interaction and different spatiotemporal expression patterns further imply that OsCBL2 and OsCBL8 have other functions.

Subsequently, we conducted further studies to determine whether OsCBL8–OsCIPK17 and OsCBL2–OsCIPK17 showed clear functional differences. OsCBL8-OsCIPK17 regulated seedlings growth, whereas OsCBL2–OsCIPK17 did not. Overexpression of *OsCBL8* and *OsCIPK17* resulted in a decrease in physiological indices, such as plant height, root length, and seedlings weight, suggesting that OsCBL8–OsCIPK17 plays a negative regulatory role in seedlings growth. Similar effects of CBL and CIPK on plant growth have been widely reported. For example, CIPK9 is involved in NH4^+^-dependent root growth in rice [33]. *CIPK3* is involved in flower development and regulates flowering time [34]. In addition, *OsCBL8* regulates seed germination. *OsCBL8* overexpression caused a significant decrease in seed germination rates compared with NIP, whereas this phenomenon was not observed with OsCBL2 and OsCIPK17. This suggests that OsCBL8 may combine with a different OsCIPK to affect germination. A similar phenomenon, in which OsCBL10 affected seed germination under waterlogging conditions, has been reported, and variations in the promoter region of *OsCBL10* influenced this regulation [35]. Overall, by measuring physiological indicators, we found that OsCBL8–OsCIPK17 regulates seedling growth. Additionally, although OsCBL8 and OsCBL2 can interact with OsCIPK17, their functions are significantly different. 

We performed RNA sequencing (RNA-seq) (Figure 4) on the parent (NIP) and overexpressed lines (*L8OE-5* and *K17OE-3*) to explore the effects of OsCBL8–OsCIPK17 on the growth of rice seedlings. We performed DEG calculations to confirm that the expression levels of *OsCBL8* and *OsCIPK17* were significantly upregulated (Figure 4A,B). There was no intersection between the upregulated and downregulated gene sets between OsCBL8 and OsCIPK17 (Figure 4C). This suggests that *OsCBL8* and *OsCIPK17* exhibit a highly consistent synergy in gene expression regulation. Since the phenotypes we obtained were low seed germination rate and seedling growth inhibition, we enriched DEGs with PO (Figure 4D,E). The results showed that the DEGs were enriched in roots, stems, leaves, flowers, and calli. This suggests that OsCBL8–OsCIPK17 may affect the growth and development of these organs, consistent with the observed phenotypic changes (Figure 4A,C,D). The DEGs were only enriched in the seeds after the overexpression of *OsCBL8*. Further research is required to unravel the regulatory role of *OsCBL8* in seed germination. Similar functions of CBL–CIPK have been previously reported. *OsCIPK3* affects flower development [34], and *OsCIPK9* regulates root growth and development [33]. Multiple CBL–CIPK modules play a role in pollen tube growth [31]. GO enrichment analysis showed that DEGs mainly play a role in the synthesis and transport of substances, regulation of enzyme activity, metabolism, response to stimulation, and reproductive development (Figure 4D,E). This suggests that OsCBL8–OsCIPK17 can regulate the above processes, as reported in previous studies. Various CIPKs regulate the growth and development of flowers and seeds [31,33,36]. For example, *GhCBL2* and *GhCIPK6* are involved in plant sugar homeostasis via interactions with the tonoplast sugar transporter TST21 [34]. In addition, we used the GSEA method, which is sensitive to non-DEGs, for enrichment analysis of KEGG. This method was also used for PO enrichment to ensure that the PO enrichment results were reliable. Overexpression of *OsCBL8* and *OsCIPK17* showed enrichment for five terms: ABC transporters, ether lipid metabolism, glycerophospholipid metabolism, phagosome proteasome, and SNARE interactions in vesicular transport. ATP-binding cassette (ABC) transporters form one of the most prominent protein families and are widespread in bacteria, archaea, and eukaryotes. They couple ATP hydrolysis to the active transport of various substrates, such as ions, sugars, lipids, sterols, peptides, and proteins [37]. Some lipids can also promote enzymatic activity [38]. Glycerophospholipids are the most abundant phospholipids in the body. As components of biofilms, glycerophospholipids are involved in protein recognition and signal transduction [39]. Phagocytosis is a process involving the uptake of relatively large particles by cells and is a central mechanism in tissue remodeling and defense against infectious agents [40]. The proteasome is a protein-destroying apparatus involved in many essential cellular functions, such as the regulation of the cell cycle, cell differentiation, signal transduction pathways, antigen processing for appropriate immune responses, stress signaling, and apoptosis [41]. SNAREs mediate membrane fusion, a fundamental process in cellular biophysics that involves viral infection, endocytosis, and exocytosis [42]. The results of the KEGG enrichment analysis showed a high degree of consistency with GO enrichment. The enrichment analysis results indicated that OsCBL8–OsCIPK17 could affect the growth of rice seedlings and their responses to external stimuli.

PPI networks were used to analyze the association of DEGs at the protein level. At the same time, we mapped part of the PO enrichment results to the interaction network to predict the impact on the rice phenotype. Many PO terms were mapped onto the PPI network. This shows that OsCBL8–OsCIPK17 participates in rice growth and development. There are many reports that CBL–CIPK is involved in the regulation of plant growth and development [31,33,36]. To understand how OsCBL8–OsCIPK17 regulates growth and development, we explored the sub-modules of the PPI network. Among them, sub-module 1, obtained after overexpression of *OsCBL8*, plays a major role in the regulation of RNA expression. The JA-responsive transcription factor JAMYB directly binds to the promoter node AGO18 in sub-module 1 to activate AGO18 transcription. AGO18 is a core RNA-silencing component that promotes rice antiviral defense by isolating miR168 and miR528, which inhibit key antiviral defense proteins [43]. This confirms that *OsCBL8* regulates AGO18 to enhance seedling disease resistance. This was confirmed by enrichment analysis. A similar phenomenon has been reported in tomatoes, whereby the CBL10–CIPK6 complex is involved in plant immune responses [14]. Sub-modules 2 and 3 revealed the involvement of chitin and Na^+^ in the seedling defense response, respectively. The sub-modules obtained after the overexpression of *OsCIPK17* showed a highly consistent function with overexpression of *OsCBL8*. That is, it can regulate the JA-mediated defense response. This implies that OsCBL8–OsCIPK17 can endow the seedlings with disease resistance. In addition, the sub-modules obtained after overexpression of *OsCIPK17* were also involved in auxin metabolism and response to temperature. Similar phenomena have been reported previously. For example, the primary function of *CaCIPK6* is in auxin transport [44]. A high expression of CIPK gene will lead to thermo-sensitive genic self-incompatible in maize [45]. *PbCBL2* is strongly induced under high temperature stress in pear. In summary, PPI network analysis demonstrated that OsCBL8–OsCIPK17 likely confers disease resistance to seedlings and regulates their growth and development. This was confirmed by the subsequent hub gene analysis.

We predicted and verified the target proteins that may have a regulatory relationship with OsCIPK17. The results showed that OsCIPK17 interacts with OsPP2C77, OsJAMYB, and OsNAC77. OsPP2C77 encodes an ABA-mediated phosphatase that interacts with CIPK [11]. ABA initially binds to its corresponding receptor protein (PYR/PYL/RCAR). Subsequently, the receptor protein binds to PP2C and mediates the PP2C dephosphorylation of CIPK, thereby regulating downstream target proteins [12]. Similar phenomena have been reported previously. For example, ABI1, ABI2, and AIP1, members of the PP2C family, can interact with CIPK to regulate downstream proteins [11,12]. 

**Figure 9:**
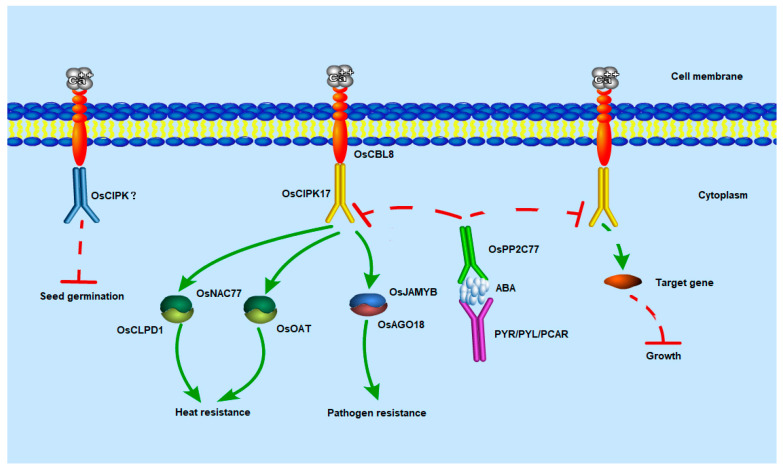
Potential regulatory mechanism of the OsCBL8-OsCIPK17 module. Calcium signaling begins with OsCBL8, which is located in the cell membrane, binds to calcium and then enforces OsCIPK17 and binds to it. OsCIPK17 may further regulate the growth and development of seedlings by interacting with some unknown target genes. This pathway is also regulated by ABA. ABA initially binds to the corresponding receptor protein (PYR/PYL/RCAR). The receptor protein with ABA further binds to OsPP2C77 and transmits the signal to it. Subsequently, OsPP2C77 dephosphorylates OsCIPK17 to inhibit its regulation of downstream targets. OsCBL8-OsCIPK17 can also endow seedlings with high temperature resistance. OsCIPK17 can phosphorylate the transcription factor OsNAC77 involved in stress response. OsNAC77 combines with the DNA-specific sequences that are located in the promoter region of OsCLPD1 and OsOAT to give seedlings the ability to resist heat stress. Meanwhile, OsCIPK17 can phosphorylate the JA-mediated transcription factor OsJAMYB. OsJAMYB can bind to the promoter of OsAGO18 to activate its transcription, so as to promote rice antiviral defense. In addition, OsCBL8 may regulate seed germination by interacting with other OsCIPK. The dotted line indicates that this process is speculative.

This interaction has only been reported in the model plant *Arabidopsis thaliana*. When the seedlings were treated with a low concentration of ABA, the overexpression line maintained the inhibition of growth compared with that in NIP. However, this inhibitory state was restored after treatment with the ABA inhibitor Na_2_WO_4_. This indicates that ABA is involved in the regulation of seedling growth by OsCBL8–OsCIPK17. There have been many reports on the involvement of ABA in the CBL–CIPK signaling pathway. In apples, CIPK22 interacts with the transcription factor AREB2 to increase sensitivity to ABA through ABA-dependent signal transduction [46]. However, in our study, the growth of seedlings after ABA or Na_2_WO_4_ treatment was inhibited compared with that under normal conditions. This is somewhat similar to the effect of auxin; that is, excess or inadequate levels of auxin will inhibit growth and development. The association of auxin with the CBL–CIPK module has been reported. In tobacco, *CaCIPK6* participates in auxin transport [44]. In addition, in PPI network analysis, node GH3.2 affects auxin metabolism. These results suggest that OsPP2C77, in response to ABA, is involved in OsCBL8–OsCIPK17-mediated regulation of seedling growth and development, which may be achieved through the auxin pathway. OsNAC77 is a plant-specific transcription factor of the NAC family that binds to the DNA-specific sequences of the CLPD1 and OAT promoters in vitro [47]. CLPD1 encodes a molecular chaperone that plays a role in heat stress responses and helps plants resist dehydration and salt stress [48]. In addition, it has been reported that overexpression of *CLPD1* in plants leads to increased tolerance to drought and salt stress [48]. *OAT* encodes an ornithine aminotransferase that enhances tolerance to drought and oxidative stress, primarily by enhancing ROS clearance and PRO pre-accumulation [49]. This suggests that OsCBL8 mediates OsCIPK17 phosphorylation of OsNAC77 to regulate seedling resistance to dehydration and salt stress. However, when rice plants are exposed to extreme temperatures and drought, they often become dehydrated. Therefore, OsCBL8–OsCIPK17–OsNAC77 likely regulates seedling responses to temperature and drought. This finding was also confirmed in the present study. Previous studies have also shown that *OsCBL8* responds to a variety of abiotic stresses, including drought [50]. Fifteen of the thirty-three *CIPK* genes (*CIPK1*, *CIPK2*, *CIPK5*, *CIPK9*, *CIPK11*, *CIPK12*, *CIPK15*, *CIPK17*, *CIPK20*, *CIPK21*, *CIPK22*, *CIPK23*, *CIPK24*, *CIPK29* and *CIPK30*) in rice are responsive to drought [7]. Further research found that overexpression of *OsCIPK12* and *OsCIPK23* can enhance drought tolerance of rice [7,51]. This study also confirmed that overexpression of *CIPK17* could increase drought tolerance of rice (Figure 8). Members of the NAC family have been widely reported to be involved in plant responses to temperature [52]. In addition, studies have shown that ABA can also participate in the regulation of NAC in plants. For example, the expression of SNAC3 is induced by drought, high salt, high temperature, ABA, and oxidative stress. It mainly improves the tolerance of transgenic plants to drought and high temperatures by scavenging ROS accumulated under stress in rice [52]. In addition, some studies have found that NAC family members regulate auxin [53]. These results indicate that OsNAC77 is likely involved in the ABA-mediated regulatory role of OSCBL8–OSCIPK17 in seedling growth and development. OsJAMYB encodes a JA-mediated transcription factor that directly binds to the AGO18 promoter to activate AGO18 transcription, thereby endowing rice with blast resistance [43]. In addition, overexpression of *JAMYB* during seed germination, seedling growth, and root elongation can increase the salt tolerance of transgenic lines [54]. These results indicate that OsCBL8–OsCIPK17 likely mediates OsJAMYB to regulate disease resistance and confers tolerance to salt stress in rice. Generally, OsCBL8–OsCIPK17 regulates seedling growth and development through ABA-mediated pathways and endows seedlings with multiple resistances (Figure 9).

## 4. Materials and Methods

Identification of gene family and construction of phylogenetic trees and Synteny analyses.

Data for identifying the gene family, construction of phylogenetic trees and synteny analyses of *CBL* and *CIPK*, with explicit annotations, were obtained from NCBI and UniProt. Similar data from the two websites were selected for subsequent analyses and experiments. MUSCLE v.3.8.31 was used to generate multiple protein sequence alignments. The aligned sequences were trimmed using TrimAL v.1.2. Phylogenetic trees were reconstructed using maximum-likelihood (ML) in IQ-TREE v.2.0 with 1000 bootstrap replicates. The optimal models of CBL and CIPK were selected automatically in IQ-TREE v.2.0 according to the Bayesian Information Criterion, set to JTT + G4 and JTT + R5, respectively. For the Bayesian inference tree, the configuration file was first created with PhyloSuite v.1.2.2 and then imported into the online website CIPRES; MrBayes v.3.2.7a was used to construct the tree. The phylogenetic trees were edited and annotated using FigTree v.1.4.3 and iTOL v.3. TimeTree was used to construct phylogenetic trees with different divergence times. A phylogenetic tree of the *CBL* gene family in rice was constructed using PhyML v.3.0, with default settings and 1000 bootstrap replicates.

The syntenic gene pairs were extracted (e-value < 1 × 10^−3^) within and between species using MCScanX, which selected *CBL* and *CIPK* gene family members, and were plotted using TBtools v.1.077. For each pair of duplicated genes, the value of nonsynonymous substitution (Ka)/synonymous substitution (Ks) was calculated using the plug-in Simple Ka/Ks calculator (NG) in TBtools v.1.077, and the frequency distribution histogram was generated using SPSS v.25. The Sankey diagram of replication types in the rice *CBL* and *CIPK* gene families was plotted using OmicStudio. Genomic data were obtained from Phytozome and Ensembl Plants.

### 4.1. Generation and Identification of Transgenic Plants

We constructed the overexpression vectors pWM101 containing the full-length coding sequences of OsCBL2, OsCBL8, and OsCIPK17. The mutant vectors for CRISPR-Cas9 were constructed by Biotechnology Co., Ltd. (Aidijing, Wuhan, China). Resistant transgenic rice plantlets, including overexpression lines and mutants, were obtained from Biotechnology Co., Ltd. The embryogenic callus of rice was infected using the *Agrobacterium*-mediated method to generate transgenic rice. Resistant transgenic rice plantlets were identified using PCR. The DNA of the T1 generation mutant lines was extracted according to the instructions provided in the DNA kit (Tiangen, Beijing, China). Primers were designed upstream and downstream of the target nucleotides to be mutated, and DNA sequencing was performed to ensure that homozygous mutant lines were obtained. The overexpression lines were identified using qPCR. The total RNA from T1 generation plants was extracted using TRIzol™ Reagent (Tiangen, Beijing, China). The RNA was converted to complementary DNA (cDNA) using a kit according to the manufacturer’s protocol. qPCR was performed using a 7500 Real-Time PCR Detection System in conjunction with the reaction Mix (Roche, Basel, Switzerland). *OsACTIN-1* (*LOC_Os05g36290*) was used as the reference gene. As shown in Supplementary Figure S20A, the mutant lines lost or gained a few nucleotides, whereas the gene expression of the overexpressed lines was significantly higher than that of Nipponbare (NIP) (Supplementary Figure S20B). All relevant experiments used the T2 generation plants. Supplementary Table S1 lists all primers used in this study.

### 4.2. Growth of Plant Materials, Gene Expression, and RNA-Seq Analysis

The growth and stress treatments of NIP were performed according to the procedure published by Lu et al. [55] with minor modifications. Seedlings were cultivated hydroponically with Mucun B nutrient solution, in a climate chamber (QY-14; Quanyou Electronic Technology Co., Ltd., Nanjing, China) at 22 °C/20 °C under a 16 h/8 h light/dark cycle at a light intensity of 100 μmol m^−2^ s^−1^. The seedlings were stressed when they reached the two real leaf stage (22 days). Stress treatments included cold or heat treatment at 8 or 39 °C, respectively, and drought treatment with 20% PEG6000, 100 mmol L^−1^ sodium chloride, and 100 μmol L^−1^ cadmium sulfate. The shoots and roots of plants subjected to all treatments were sampled, frozen in liquid nitrogen, and stored at −80 °C for gene expression analysis. The seedlings used for measuring plant height, root length, fresh weight, and dry weight were cultured for 2 weeks. Germination rate was calculated after culturing for 1 week. Transcriptome sequencing was performed after culturing for 2 weeks. The samples were sent to Yongji Biotechnology Co., Ltd. (Guangzhou, China) and sequenced using Illumina NovaSeq 6000. Three biological replicates of each line were sequenced. The original RNA-seq data were submitted to the National Genomics Data Center (https://ngdc.cncb.ac.cn/); access number is PRJCA009668 and this BioProject will be available on 19th May 2024. Seedlings were cultured in the same manner for experiments involving abscisic acid (ABA), Na_2_WO_4_ (an inhibitor of ABA synthesis), and temperature responses. The high-temperature treatment was performed at 39 °C/30 °C instead of 22 °C/20 °C.

Gene expression was analyzed using RT-qPCR as described in the previous section. The relative expression levels were calculated according to Cao et al. [56] and visualized as a heat map using TBtools v.1.077. In addition, heat map data were obtained from BAR.

### 4.3. Subcellular Localization Analysis

Tobacco seeds were surface-sterilized with 70% ethanol and 20% bleach. They were cultured for 4 weeks in a climate chamber. The coding sequences (CDS) of *OsCBL2, OsCBL8*, and *OsCIPK17* were inserted into the enhanced green fluorescent protein (eGFP, pCAMBIA-2300-35S-N-eGFP-OCS) vector to generate fusion constructs, which were then transferred into *Agrobacterium* (GV3101) and co-cultured to infect tobacco. The infected tobacco was observed after dark culture for 3 days. All images were captured using a confocal laser microscope of TCS SP8 (Leica, Heidelberg, Germany).

### 4.4. Production of Transgenic Arabidopsis thaliana and GUS Tissue Staining

Transgenic *Arabidopsis thaliana* with promoters of the *OsCBL8*, maintained in this study, was produced following the methods described above [26]. β-Glucuronidase (GUS) staining was performed in tissues of T2 transgenic *Arabidopsis thaliana* containing the GUS reporter gene using a kit (Tiangen, Beijing, China).

### 4.5. Yeast Two-Hybrid and Bimolecular Fluorescent Complementation (BiFC) Assays 

Yeast two-hybrid (Y2H) assays were performed using the Matchmaker Gold Yeast Two-Hybrid System (Clontech, Kusatsu, Japan), according to the manufacturer’s instructions. Full-length coding sequences of *OsCBL* were cloned into the pGADT7 vector, and those of *OsCIPK* were introduced into the pGBKT7 vectors. The plasmids were co-transformed into the Y2HGold strain using the lithium acetate method, according to the TRANFOR protocol. The transformed yeast cells were grown on DDO (SD-Leu/-Trp) medium for 3–5 days. Positive colonies were picked up and grown on TDO (SD-Leu/-Trp/-His) and QDO (SD-Leu/-Trp/-His/-Ade) media for another 3–5 days. 

Full-length coding regions of *OsCBL* and *OsCIPK* were cloned into vectors pSPYCE or pSPYNE [57]. BiFC assays were performed according to the previously described method [58]. The tested construct pairs were expressed in the leaves of *Nicotiana benthamiana* for three days. YFP fluorescence in the transformed leaves was analyzed using a confocal laser microscope (TCS SP8, Leica, Heidelberg, Germany).

### 4.6. Analysis of Physiological Parameters 

Seed vigor was measured using 2, 3, 5-triphenyltetrazolium chloride (TTC) staining. Shelled seeds were placed in a 2% TTC staining solution and placed in a 33 °C water bath for 2 h for observation. Reactive oxygen species (ROS) content was detected using the fluorescent probe H2DCFDA according to the manufacturer’s instructions. Analysis of proline and superoxide dismutase was performed using a kit (Jiancheng, Nanjing, China) according to the manufacturer’s instructions and measured using a microplate detector (EnSpireTM 2300, PerkinElmer, Waltham, MA, USA) and ultraviolet spectrophotometer (Evolution 300 BB, ThermoFisher, Waltham, MA, USA).

### 4.7. Measurement of Hormone Content

Leaves of the 2-week-old seedlings of the NIP and overexpression lines were selected for hormone detection. The samples were stored in dry ice and forwarded to PANOMIX (Suzhou, China) for hormone detection using liquid chromatography–mass spectrometry. Three biological replicates were detected for each line.

### 4.8. Enrichment Analysis of Differential Genes

After obtaining the original transcriptome sequencing data, we used FastQC and Trimmomatic for quality monitoring and control to ensure that they could be used for subsequent analyses. Kallisto was used to obtain the gene expression matrix. The transcription level matrix obtained by Kallisto was transformed into a gene-level matrix using Trans Value Sum. Differentially expressed genes (DEGs) were detected using DESeq2. PlantGSEA was used for enrichment analysis, and the results were visualized using OmicStudio. Fisher’s exact test was used for statistical analysis and the Benjamini–Yekutieli procedure (FDR under dependency) for multi-test adjustment. Gene set enrichment analysis was performed using GSEA v.4.1.0, with default settings. Volcanic maps and other data were visualized in the same manner.

### 4.9. Construction of the Protein–Protein Interaction (PPI) Network

The acquired DEGs were introduced into STRING to determine interactions. Genetic interactions were introduced into Cytoscape v.3.8.2 to construct the network. The plug-in MCODE with default settings was used to obtain the sub-modules. The expression levels of related genes were introduced into OmicStudio, and correlation analyses of genes in the sub-modules were carried out. Finally, the cytoHubba plug-in with default settings was used to identify hub genes in the network. The results were displayed in the form of a heat map using TBtools v.1.077.

### 4.10. Statistical Analysis

Figure legends describe in detail the experiments, statistical analyses, and the number of samples. Data analysis and visualization were performed using GraphPad Prism v.9.0.0.

## 5. Conclusions

We found that OsCBL8 negatively regulated seed germination and interacted with OsCIPK17 to regulate seedling growth. OsPP2C77 further regulates this process via the ABA signaling pathway. Additionally, OsCBL8 can mediate the phosphorylation of OsNAC77 and OsJAMYB by OsCIPK17, thus providing rice with resistance to high temperatures and drought and helping it resist pathogens.

## Data Availability

The raw sequence data have been deposited in National Genomics Data Center, China National Center Bioinformation/Beijing Institute of Genomics, Chinese Academy of Sciences (BioProject: PRJCA009668) that are publicly accessible at https://ngdc.cncb.ac.cn/.

## Supplementary Materials

The following supporting information can be downloaded at: https://www.mdpi.com/article/10.3390/ijms232012451/s1.
