# Supplementary material for: The OsCBL8–OsCIPK17 Module Regulates Seedling Growth and Confers Resistance to Heat and Drought in Rice
Source: Int J Mol Sci. 2022 Oct 18;23(20):12451. doi: 10.3390/ijms232012451 (PMC9604039; doi:10.3390/ijms232012451)
Supplement: Supplementary file 1 [file ijms-23-12451-s001.zip › ijms-1910120-supplementary.pdf]

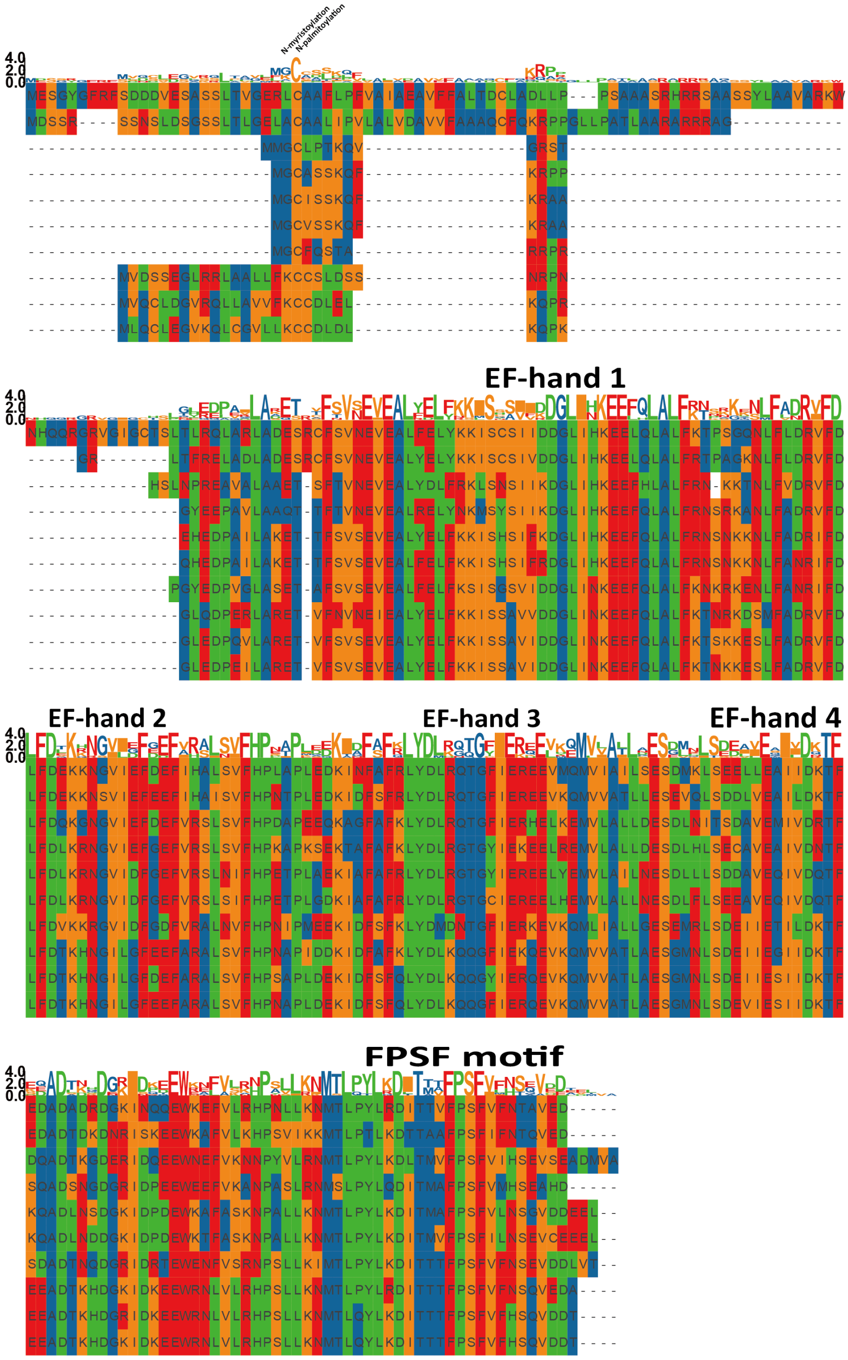

Supplemental Figure. S1. Multiple sequence alignment of rice CBL protein with conserved motifs. Myristoylation sites and palmitoylation sites are shown at the N-terminal. FPSF motif is shown at the C-terminal. There are four EF-hand domains in the middle part of the proteins.

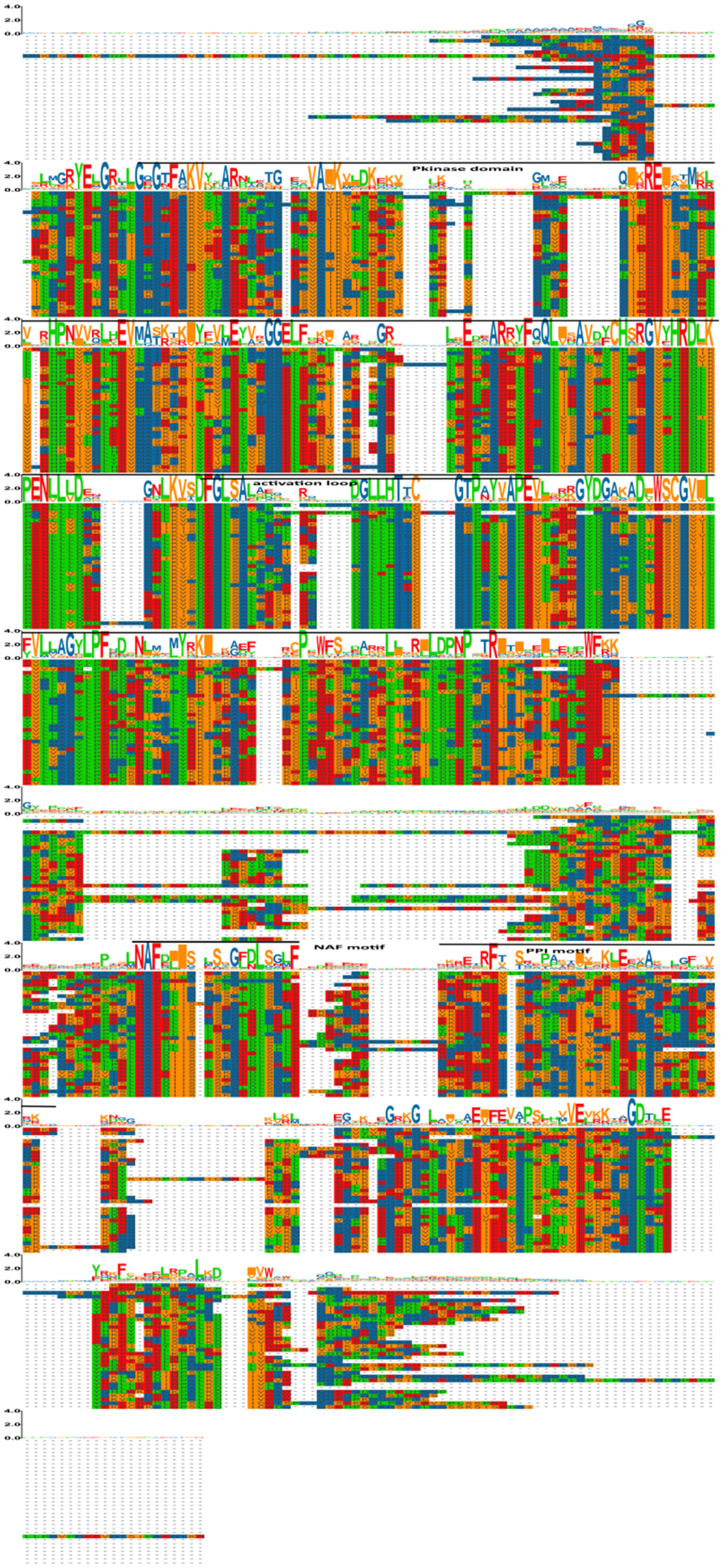

Supplemental Figure. S2. Multiple sequence alignment of rice CIPK protein with conserved motifs. The kinase domain is displayed at the N-terminal and has a conserved activation loop. The NAF motif and PPI motif are displayed at the c-terminal.

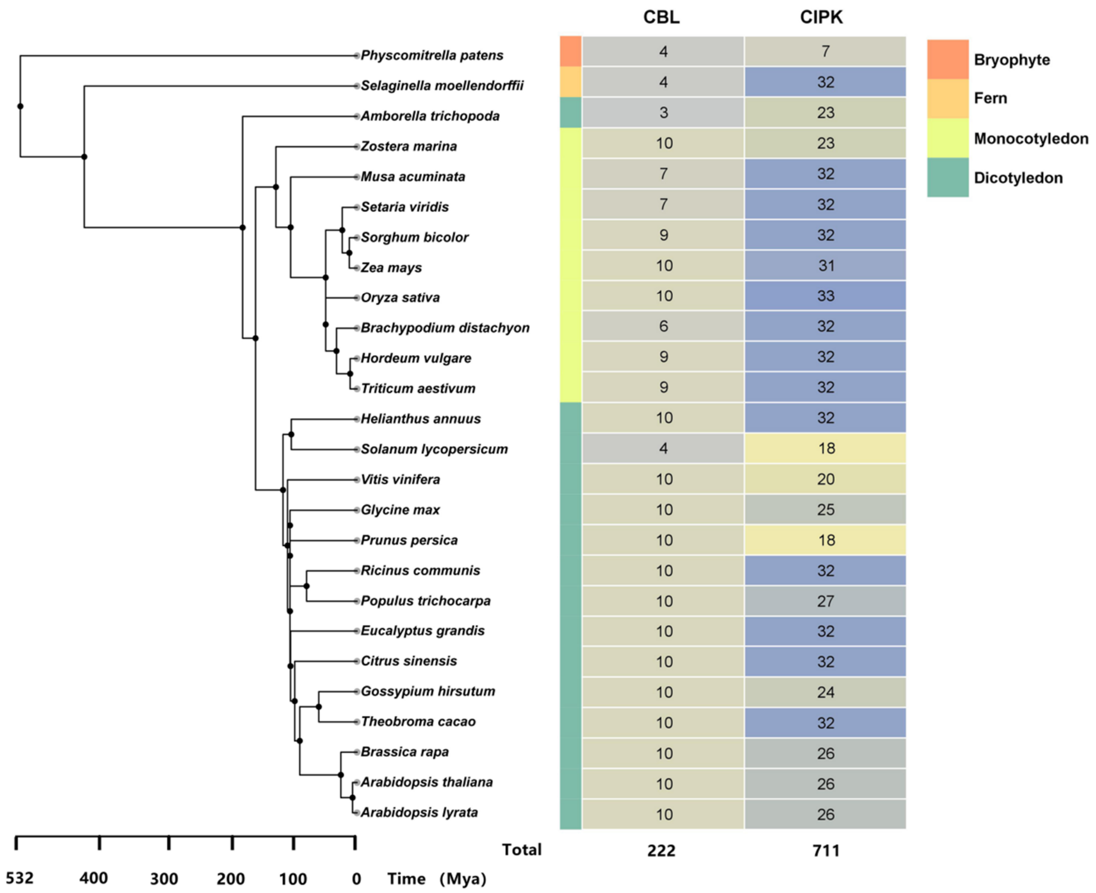

Supplemental Figure. S3. Statistics of CBL and CIPK in different plant species. The phylogenetic tree on the left shows the divergence time of 26 different species selected from monocotyledons, dicotyledons, bryophyte and ferns. The heat map on the right shows the number of members from *CBL* and *CIPK* gene families in different plants.

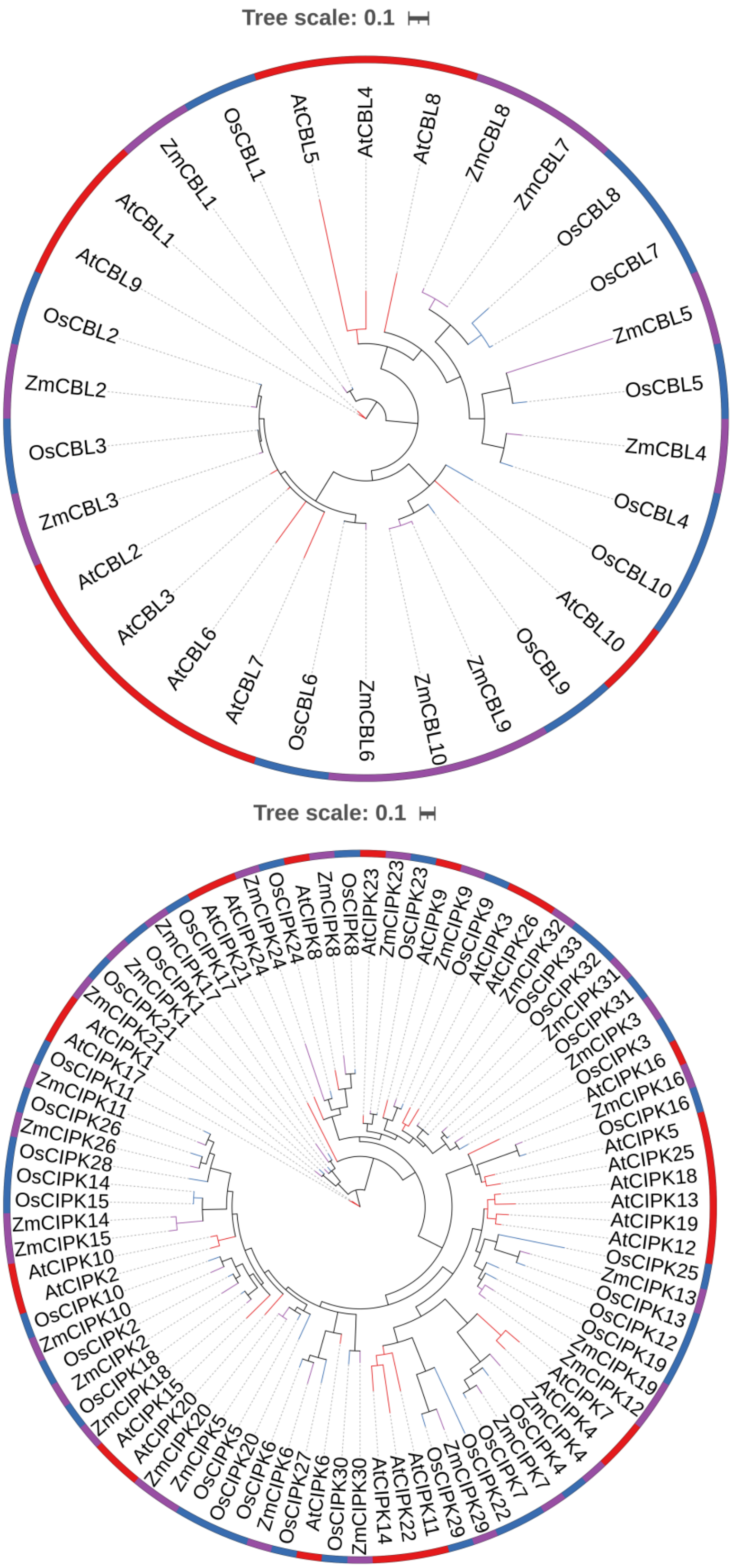

Supplemental Figure. S4. Maximum-likelihood gene tree topology for the *CBL* and *CIPK* gene family. Red for Arabidopsis, blue for rice and purple for corn.

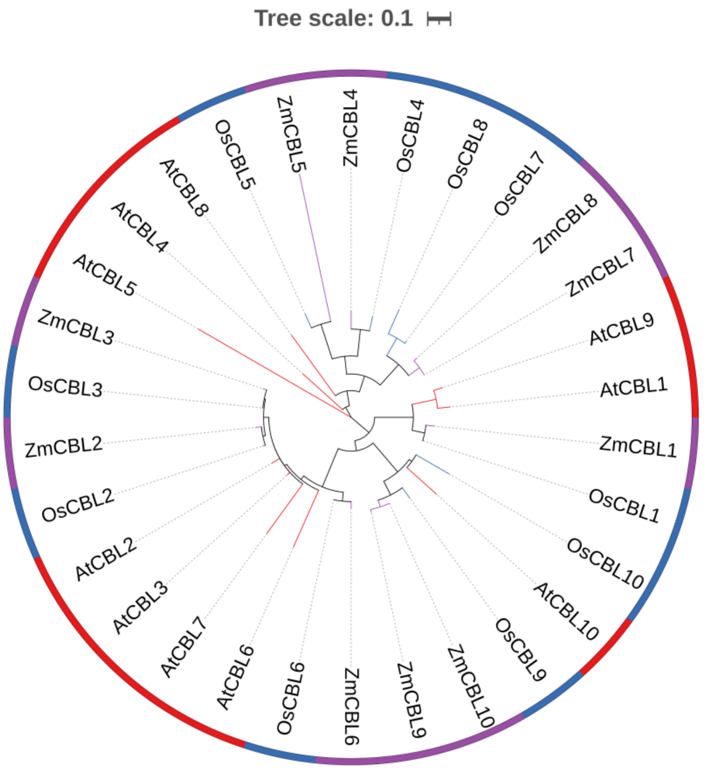

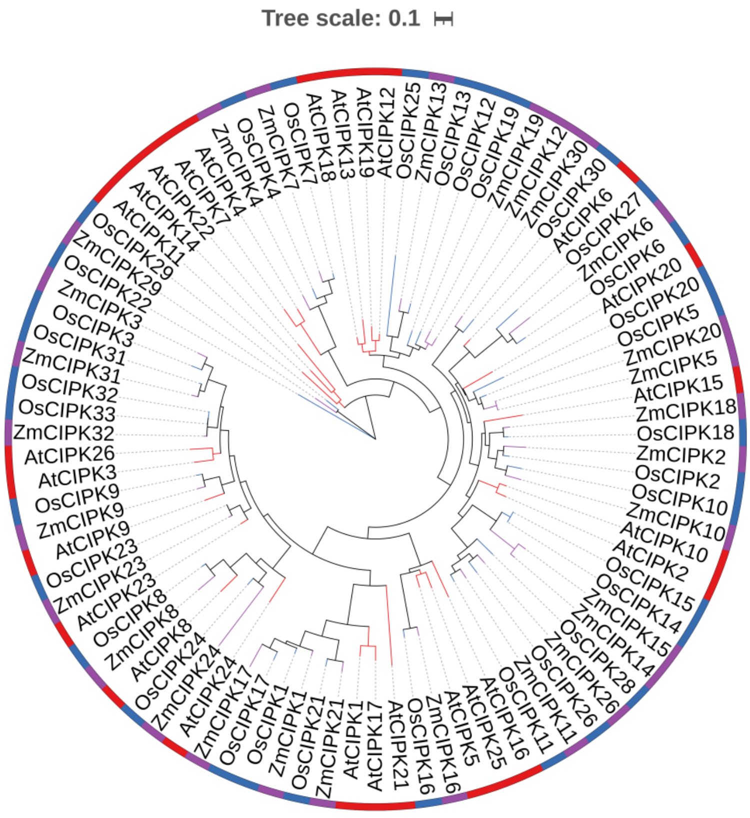

Supplemental Figure. S5. Bayesian inference gene tree topology for the *CBL* and *CIPK* gene family. Red for Arabidopsis, blue for rice and purple for corn.

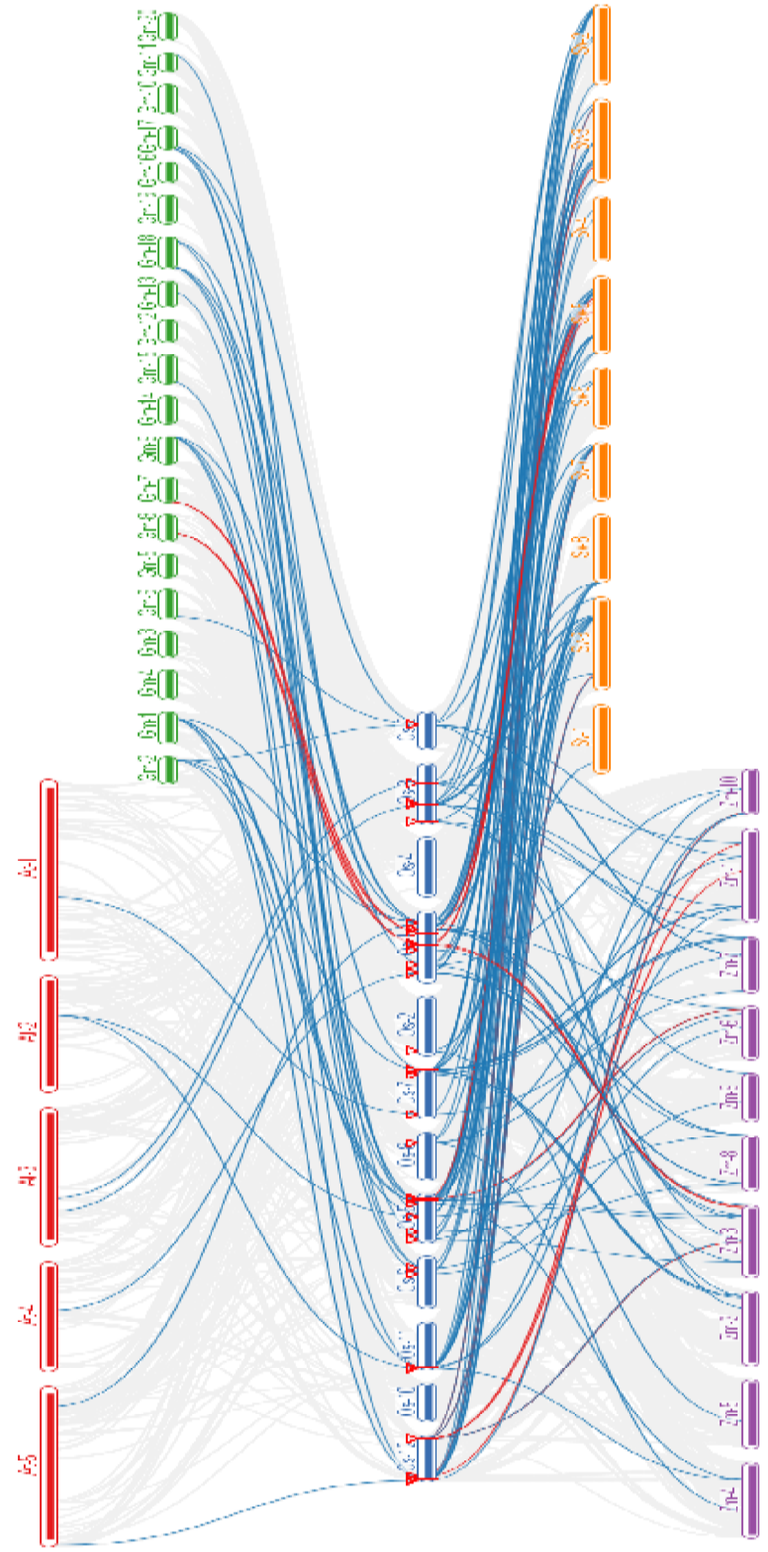

Supplemental Figure. S6. Syntenic relationships of the *CBL* and *CIPK* gene family in rice (Os), maize (Zm), setaria (Sv), soybean (Gm), and Arabidopsis (At). Red lines represent the *CBL* family, and blue lines represent the *CIPK* family. Red triangles indicate the position of *CBL* and *CIPK* genes on the rice chromosome.

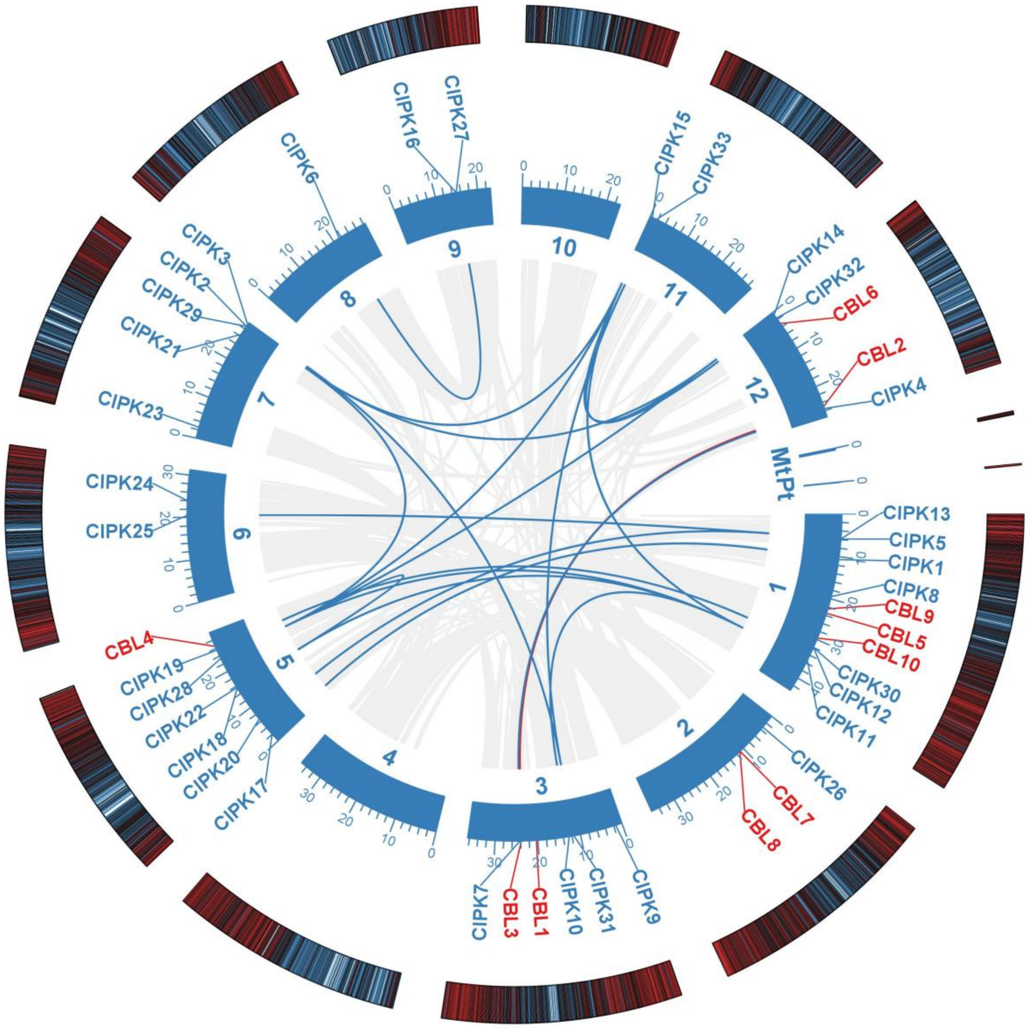

Supplemental Figure. S7. Syntenic analysis of the *CBL* and *CIPK* gene families in rice. Gene density is also added to the graph. Red indicates high gene density, while blue is the opposite.

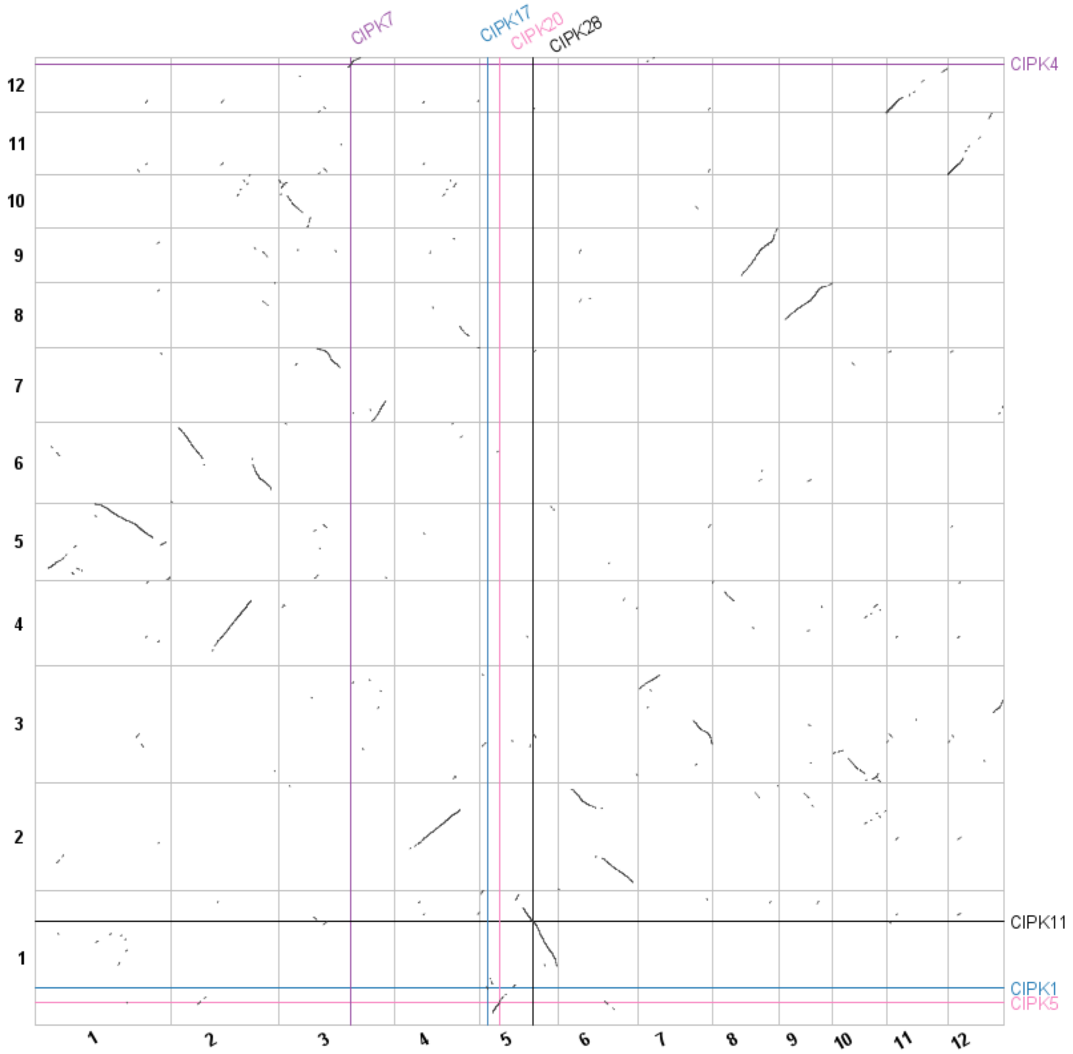

Supplemental Figure. S8. Dot plot image of rice genome-wide synteny. Gene-pairs experiencing WGD are represented by lines of the same color.

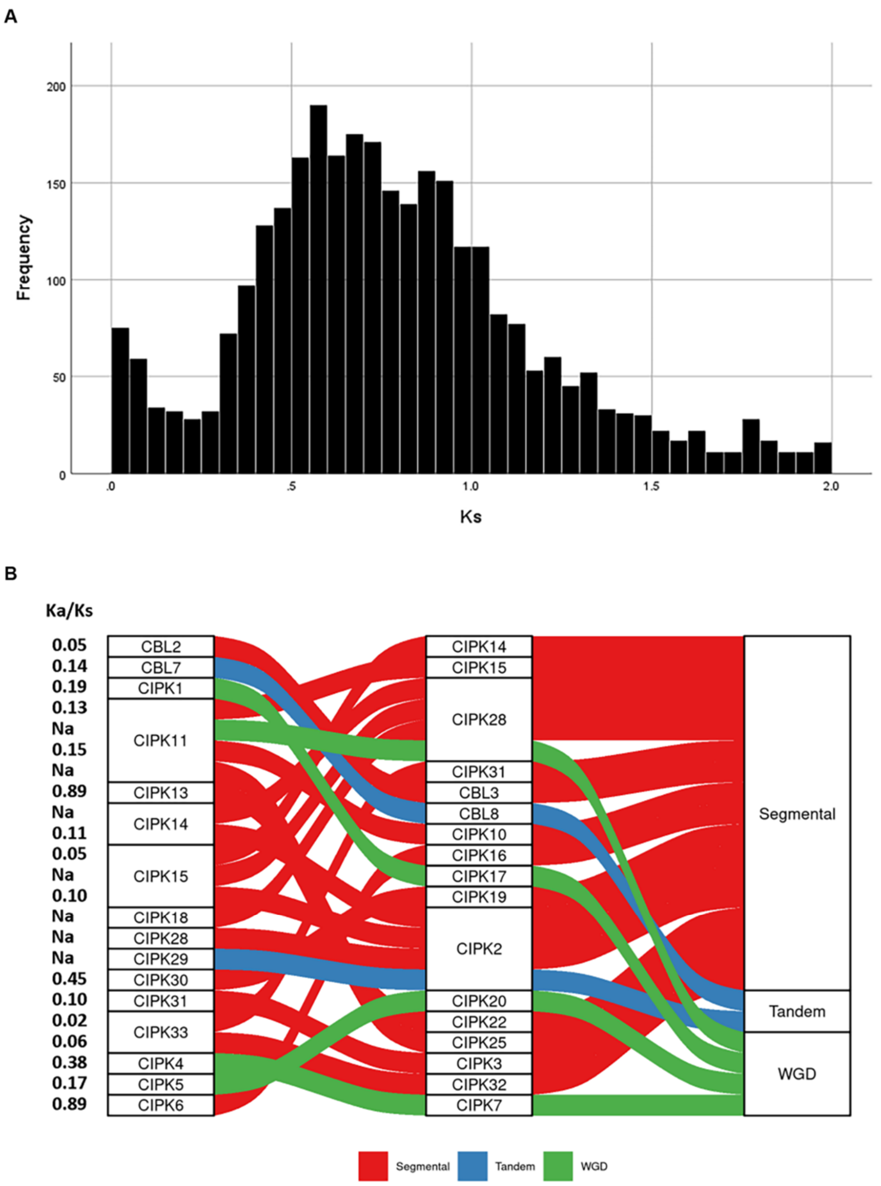

Supplemental Figure. S9. Syntenic analysis of the *CBL* and *CIPK* gene families in rice. (A) Ks frequency distribution map of gene-pairs with synteny relationship in rice genome. (B) Sankey diagram of replication types in rice *CBL* and *CIPK* gene families. Na indicates that it cannot be calculated due to the influence of high sequence divergence value (pS>=0.75).

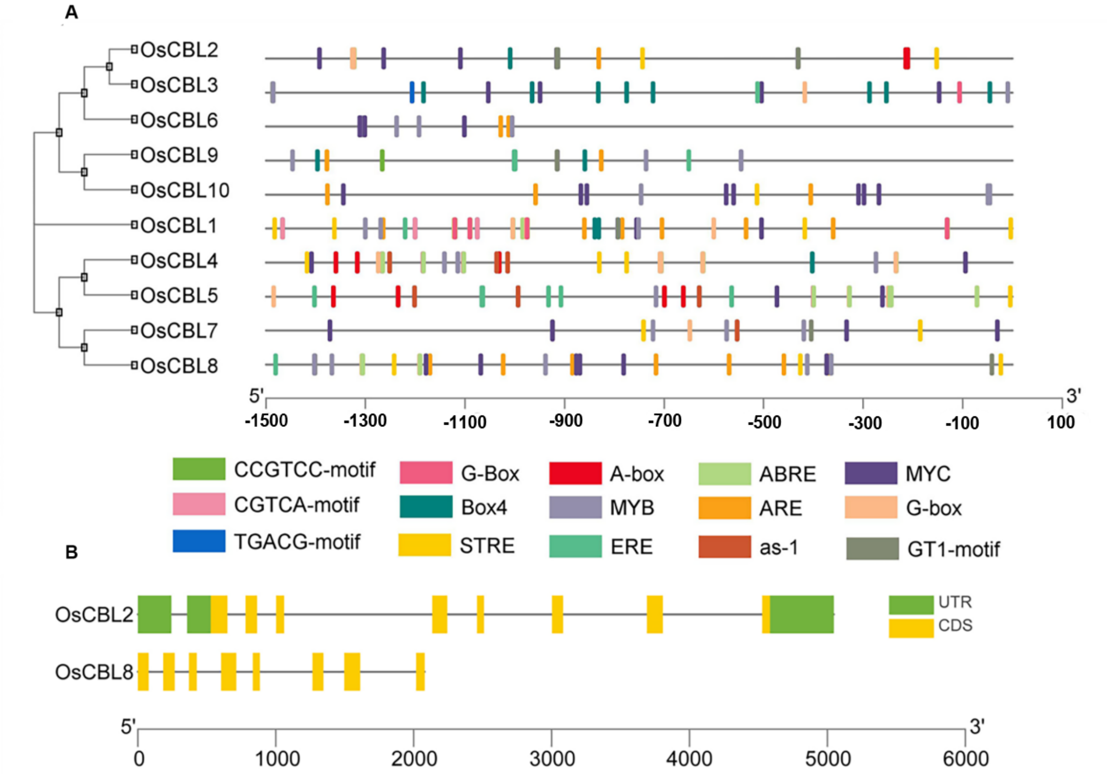

Supplemental Figure. S10. Promoter analysis of rice *CBL* gene family and gene structure display of *OsCBL2* and *OsCBL8*. (A) Prediction of promoter distribution in 1500 bp upstream region of *OsCBL* with maximum-likelihood gene tree topology. (B) Gene structure display of *OsCBL2* and *OsCBL8*.

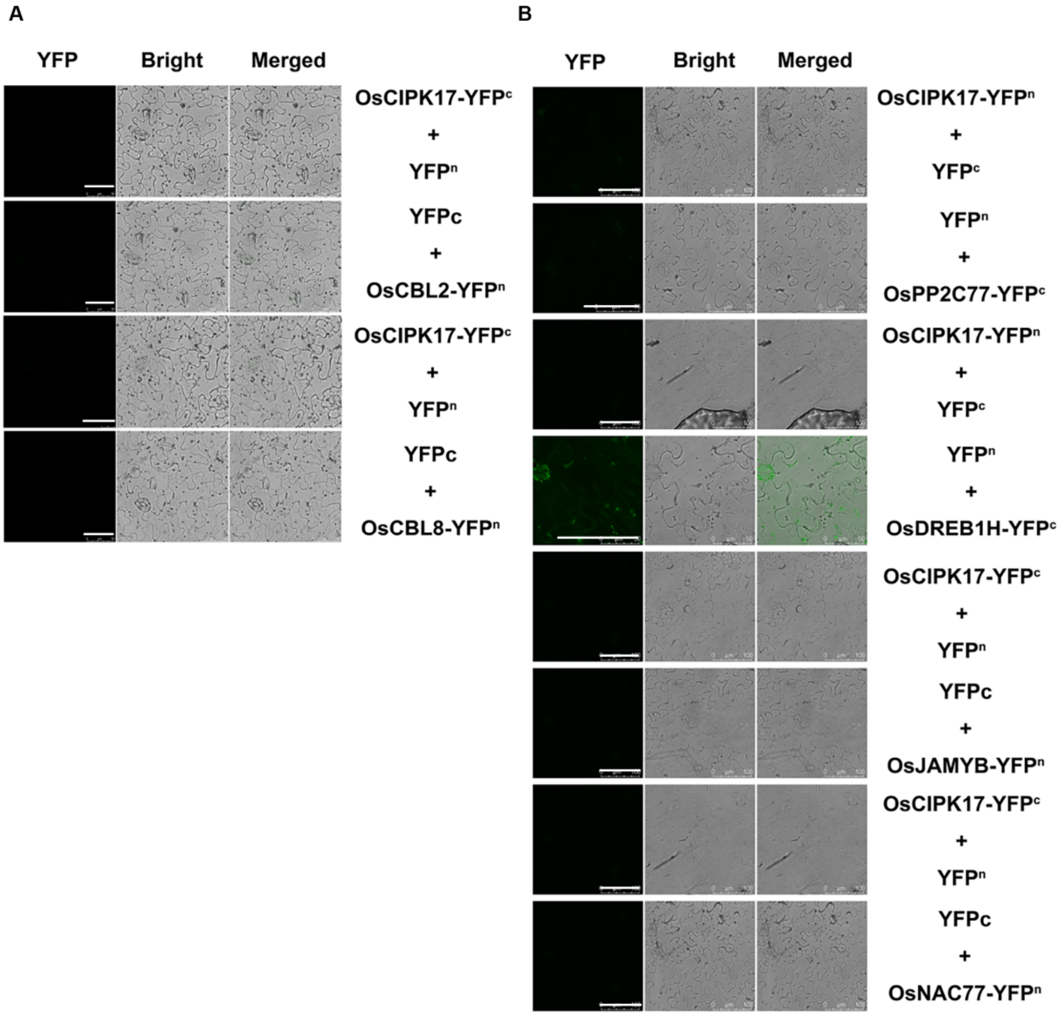

Supplemental Figure. S11. Images of BIFCcontrols. (A) Controls of firuge 1B. Bar = 100 μm. (B) Controls of firuge 6F. Bar = 100 μm.

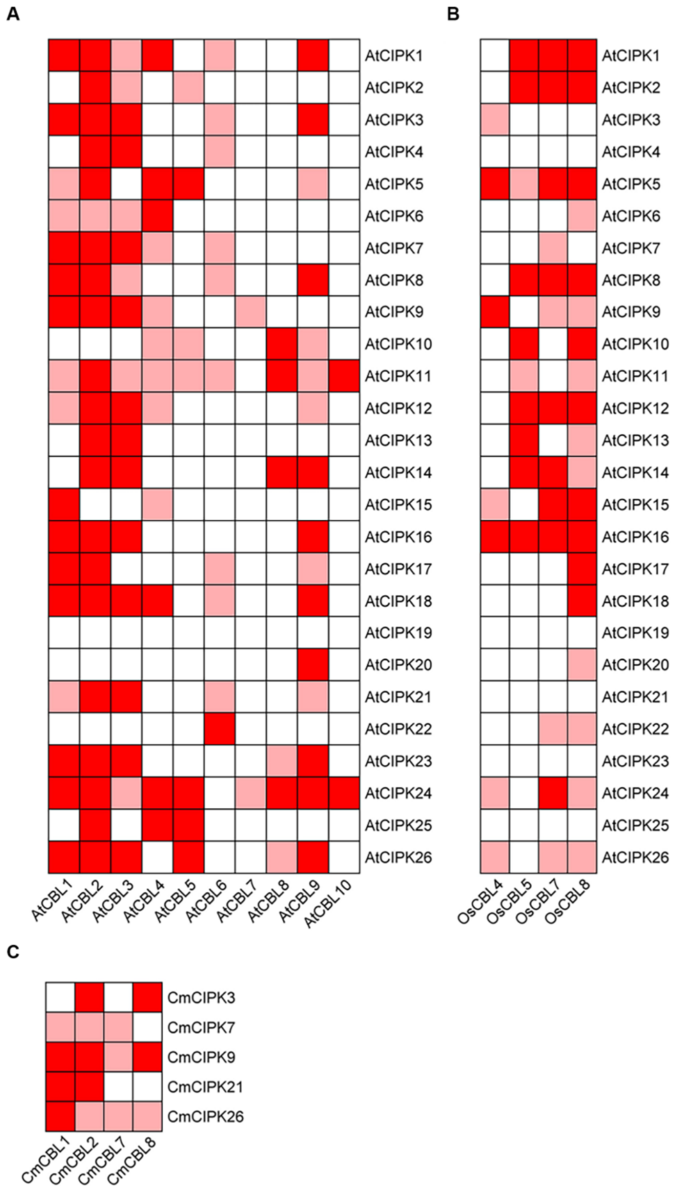

Supplemental Figure. S12. Specific interaction between CBL and CIPK. (A) The figure shows the interaction between CBL and CIPK in *Arabidopsis*. Deep red indicates strong interaction, light red indicates weak interaction, and white indicates no interaction. (B) The figure shows the interaction between CBL from rice and CIPK from *Arabidopsis*. Deep red indicates strong interaction, light red indicates weak interaction, and white indicates no interaction. (C) The figure shows the interaction between CBL and CIPK in pumpkin. Deep red indicates strong interaction, light red indicates weak interaction, and white indicates no interaction.

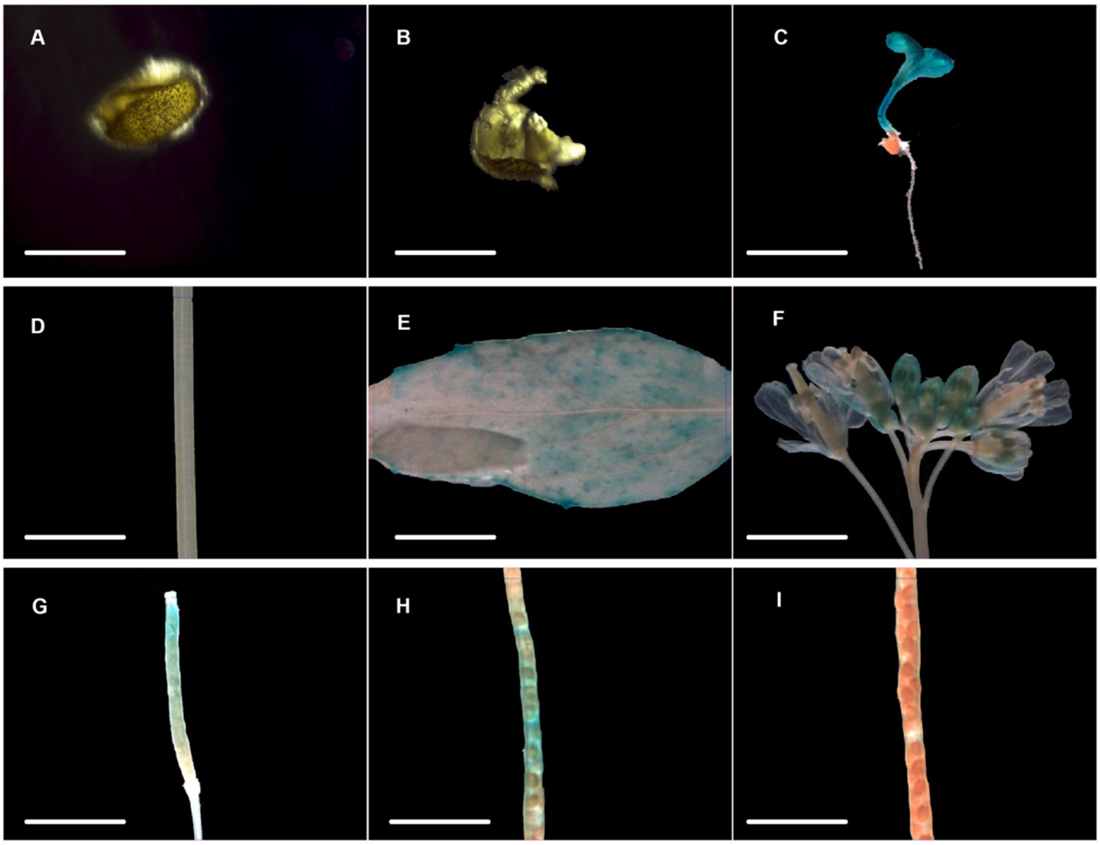

Supplemental Figure. S13. GUS tissue staining of *Arabidopsis thaliana* transformed with the promoter of *OsCBL8*. A. Seed; B. Seed embryo; C. One-week-old seedlings; Stems of one-month-old plants; E. Stem leaf of one-month-old plants; F. Flowers of one-month-old plants; G. H, I, pods of different parts of one-month-old plants. The scale of seed and embryo is 2 mm; The rest of the scales are 200 μm.

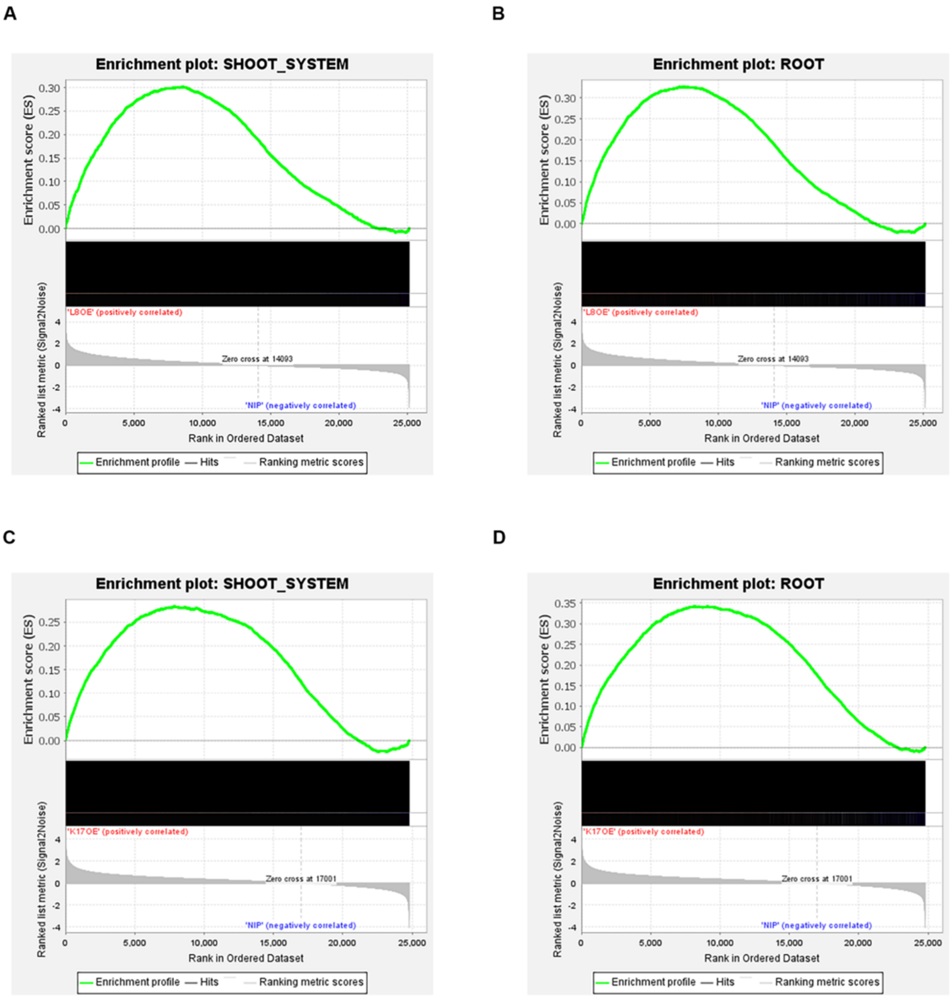

Supplemental Figure. S14. Gene Set Enrichment Analysis based on PO database. (A) GSEA in shoot system for *OsCBL8* overexpression lines. Enrichment Score (ES) = 0.30162418, Normalized Enrichment Score (NES) = 1.0771977, p-value = 0, q-value = 0. (B) GSEA in root for *OsCBL8* overexpression lines. ES = 0.3265098, NES = 1.1673537, p-value = 0, q-value = 0. (C) GSEA in shoot system for *OsCIPK17* overexpression lines. ES = 0.28341126, NES = 1.093442, p-value = 0, q-value = 0. (D) GSEA in root for *OsCIPK17* overexpression lines. ES = 0.34221265, NES = 1.3186401, p-value = 0, q-value = 0.

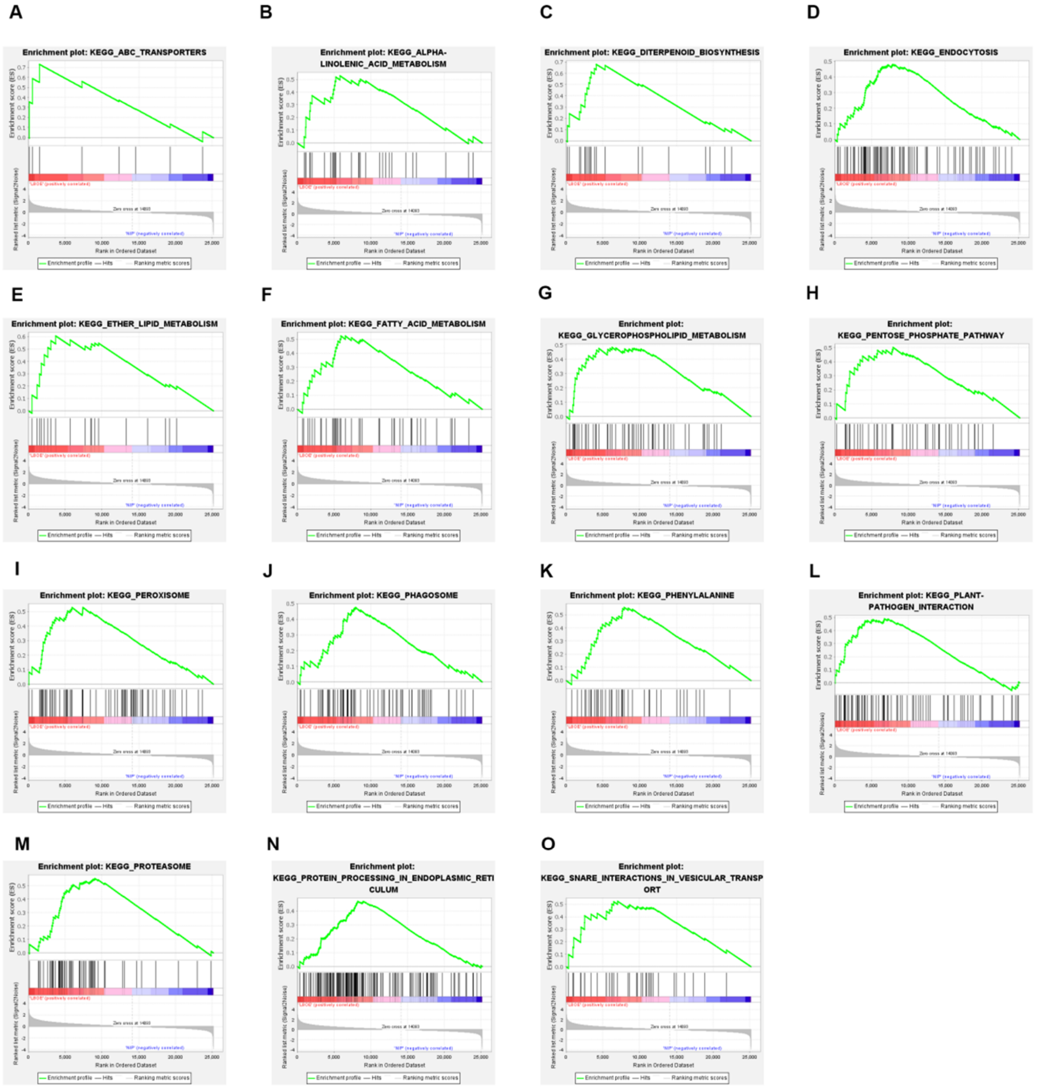

Supplemental Figure. S15. Gene Set Enrichment Analysis for *OsCBL8* overexpression lines based on Kyoto Encyclopedia of Genes and Genomes (KEGG) database. The specific data of ES, NES, p-value and q-value are shown in the table (Supplemental Table S2).

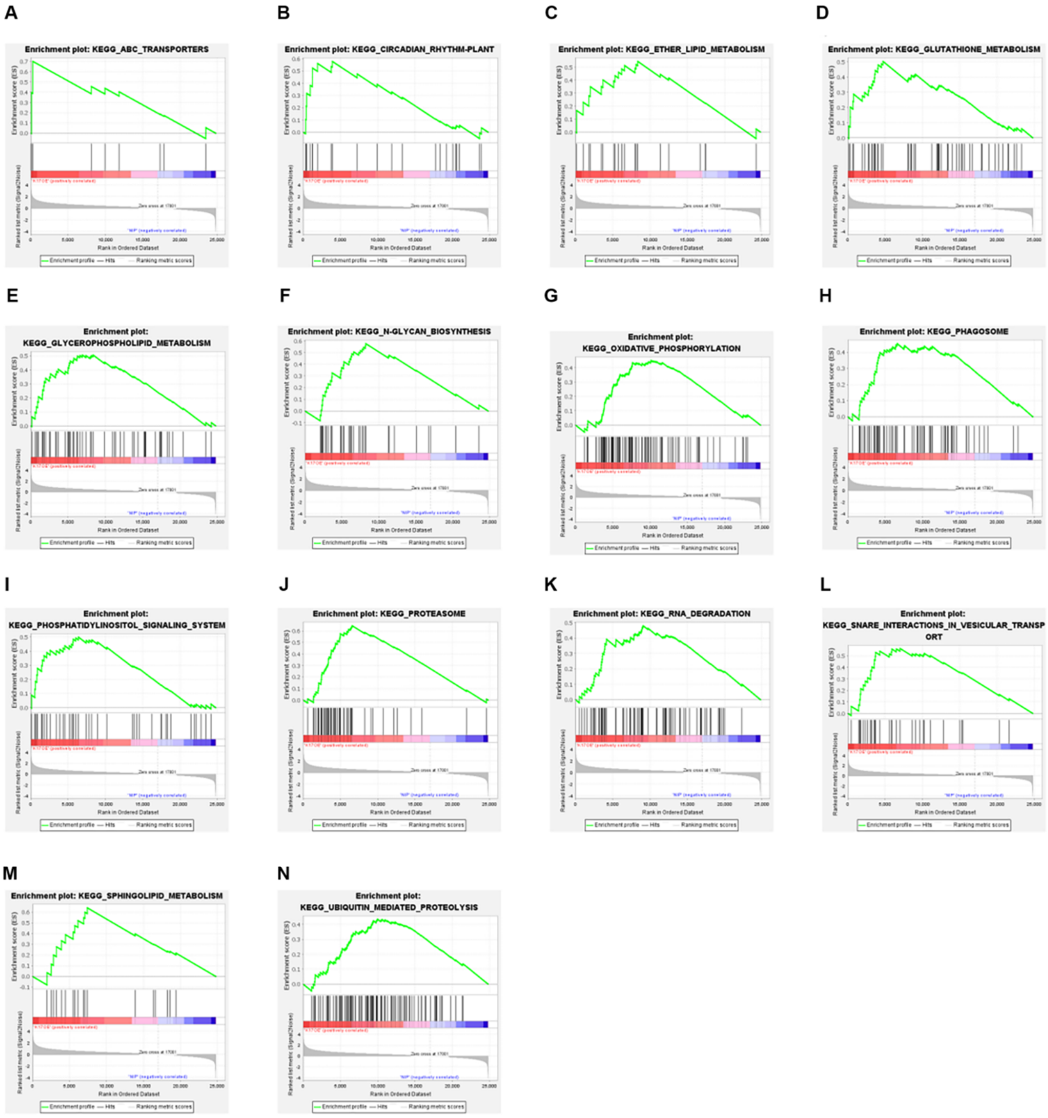

Supplemental Figure. S16. Gene Set Enrichment Analysis for *OsCIPK17* overexpression lines based on KEGG database. The specific data of ES, NES, p-value and q-value are shown in the table (Supplemental Table S3).

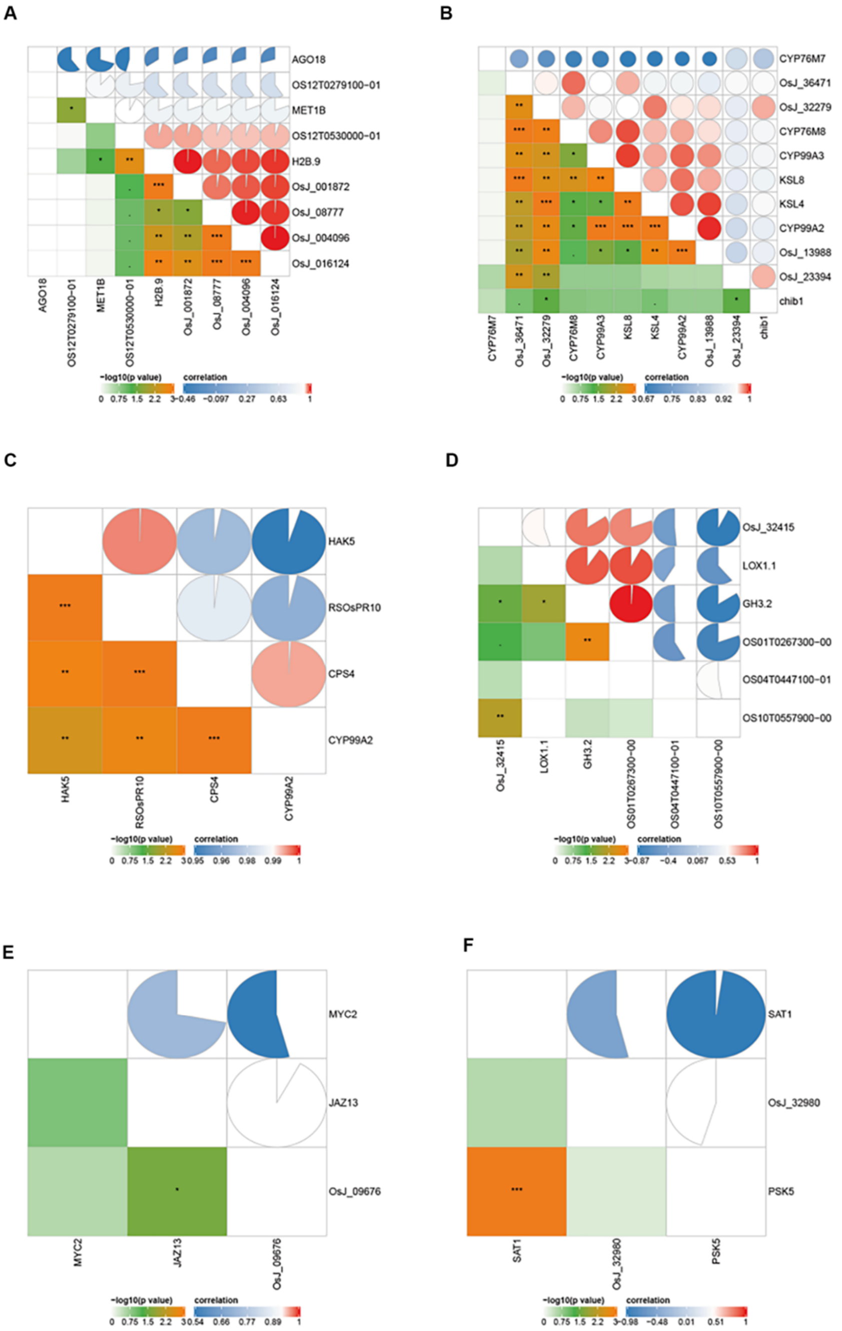

Supplemental Figure. S17. Correlation analysis of sub-modules in the PPI network. (A), (B), (C) correspond to sub-modules 1, 2 and 3 after overexpression of *OsCBL8*, respectively. (D), (E), (F) correspond to sub-modules 1, 2 and 3 after overexpression of *OsCIPK17*, respectively. *P < 0.05, **P < 0.01, ***P < 0.001.

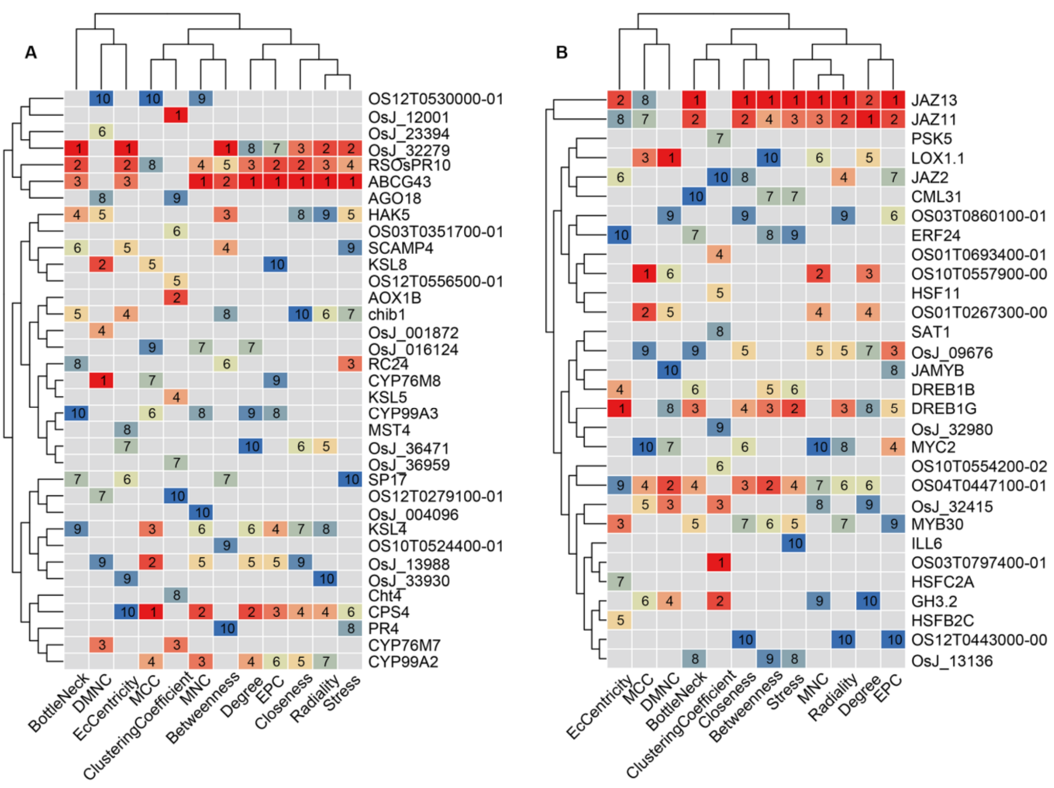

Supplemental Figure. S18. Hub genes selected by different methods. (A) and (B) Hub genes generated from DEGs of lines overexpressing *OsCBL8* and *OsCIPK17*. They are ranked according to their scores. The higher the score, the higher the ranking (red). Only the top ten genes are show

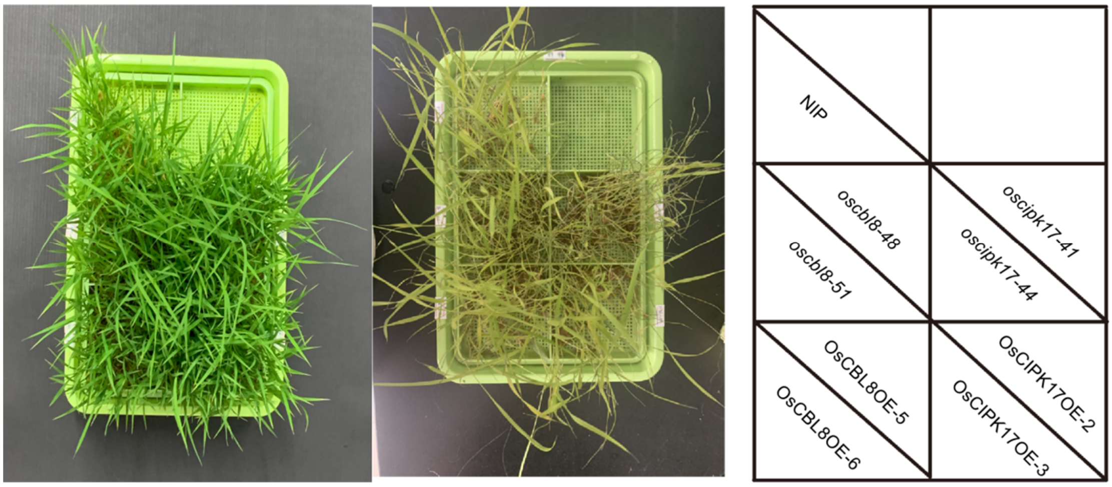

Supplemental Figure. S19. Phenotype of 2-week-old seedlings after 9 days of high temperature treatment. The left is the plant under normal conditions, the middle is the plant under high temperature treatment, and the right is the schematic diagram of plant planting location.

A

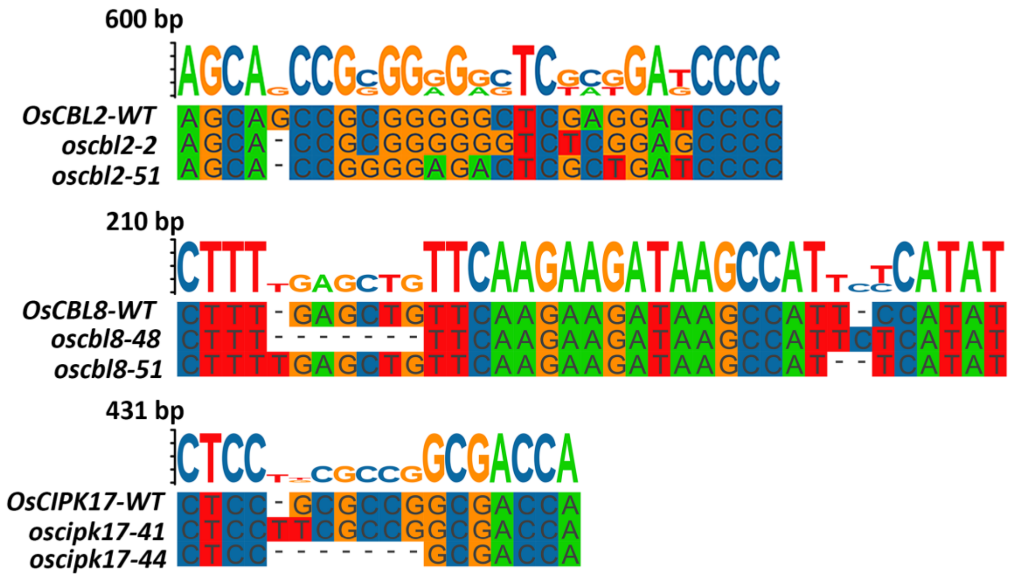

B

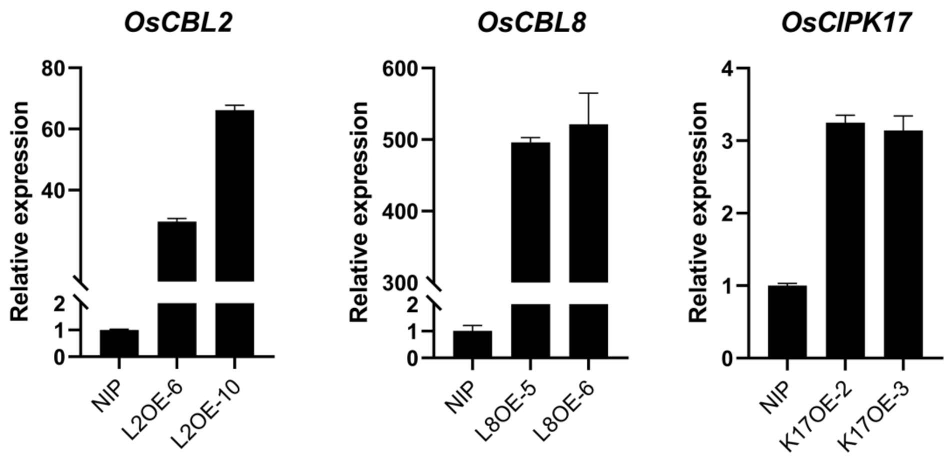

Supplemental Figure. S20. Identification of mutant and overexpression lines. (A) Multiple sequence alignment results of mutation sites in different lines. These results were obtained by DNA sequencing. (B) The gene expression of overexpressed lines was verified by qPCR. Data are mean ± SD from 3 biological replicates.

Supplemental Table S1. Primers used in this study.

Gene	Access number	Sequence (5'-3')	Vector/Usage
OsCBL2	*LOC_Os12g40510*	F:CTGATCTCAGAGGAGGACCTGCATATGGTGCAGTGTCTCGACGGG	pGBKT7
		R:GGCCGCTGCAGGTCGACGGATCCCGGTGTCATCGACCTGGGAATG	
OsCBL8	*LOC_Os02g18930*	F:CTGATCTCAGAGGAGGACCTGCATATGGGCTGTGTATCATCAAAGCAG	pGBKT7
		R:GGCCGCTGCAGGTCGACGGATCCCCAACTCTTCCTCACAAACTTCAGA	
OsCIPK8	*LOC_Os01g35184*	F:TACGACGTACCAGATTACGCTCATATGGTGGGCGGAGGGGCG	pGADT7
		R:TGCAGCTCGAGCTCGATGGATCCCGCGCTTCGATAGCCGCGA	
OsCIPK17	*LOC_Os05g04550*	F:TACGACGTACCAGATTACGCTCATATGGTGAAGGGAGGGAGGGAG	pGADT7
		R:TGCAGCTCGAGCTCGATGGATCCCCAAAGCGAACAGCGGCGT	
OsCIPK23	*LOC_Os07g05620*	F:TACGACGTACCAGATTACGCCATATGAGCGTGTCGGGCGGG	pGADT7
		R:TGCAGCTCGAGCTCGATGGATCCCCGGTGACCTCCGATGCTG	
OsCIPK24	*LOC_Os06g40370*	F:TACGACGTACCAGATTACGCTCATATGGGAGGGGAGGAGGGAATG	pGADT7
		R:TGCAGCTCGAGCTCGATGGATCCCGCATGTGGCTGTCCTCAG	
OsCIPK27	*LOC_Os09g25100*	F:CATGGAGGCCGAATTCCCGGGATGGAGGGGAAGGGGGTG	pGADT7
		R:CTAGTTATGCGGCCGCTGCAGTCAGGTCGCCGCCGGCGA	
OsCBL2	*LOC_Os12g40510*	F:GCGACTAGTATGGTGCAGTGTCTCGACGG	pSPYNE
		R:GCGCTCGAGGGTGTCATCGACCTGGG	
OsCBL8	*LOC_Os02g18930*	F:GCGACTAGTATGGGCTGTGTATCATCAAAGCAG	pSPYCN
		R:GCCTCGAGCAACTCTTCCTCACAAACTTCAGAG	
OsCIPK17	*LOC_Os05g04550*	F:GCGACTAGTATGGTGAAGGGAGGGAGGGA	pSPYCE
		R:GCGCTCGAGCAAAGCGAACAGCGGC	
OsCBL8	*LOC_Os02g18930*	F: GGTACCATGGGCTGTGTATCATCAAAGC	PWM101
		R:TCTAGATTACAACTCTTCCTCACAAACTTCAG	
OsCIPK17	*LOC_Os05g04550*	F: CCATGGAGATGGTGAAGGGAGGGAGGGA	PCM1307
		R: ACTAGTCAAAGCGAACAGCGGC	
OsCBL8	*LOC_Os02g18930*	F:AAACGCCTTTGGGAGACAAG	qPCR
		R:AGAGCAAGCACCATTTCGTG	
OsCIPK17	*LOC_Os05g04550*	F:CAGATCCCAAAGTGGCTTTCAC	qPCR
		R:GTGTGATTTGATCCCTGCCATG	
OsACTIN-1	*LOC_Os05g36290*	F:CTTCATAGGAATGGAAGCTGCGGGTA	qPCR
		R:CGACCACCTTGATCTTCATGCTGCTA	
OsCBL8	*LOC_Os02g18930*	F: GGGCTGTGTATCATCAAAGCAGTTC	Mutant identification
		R:GTAGAACTGTGCTTCTTCAGATAATGCC	
OsCIPK17	*LOC_Os05g04550*	F:GCCAATCCGTCTTCCATTATATTTCTC	Mutant identification
		R:GGAGGCGAACCACGCAATTT	
OsAKI2	LOC_Os03g63940	F:CTGATCTCAGAGGAGGACCTGCATATGTTTTCCCACGGCGCT	pGBKT7
		R:GGCCGCTGCAGGTCGACGCATCCCTTAGCTCAGCAAGAACTTGAAAATATC	
OsDREB1B	LOC_Os09g35010	F:CTGATCTCAGAGGAGGACCTGCATATGGAGGTGGAGGAGGCGGCG	pGBKT7
		R:GGCCGCTGCAGGTCGACGGATCCCTTAGTAGCTACAGAGCGGCATGTCGGA	
OsHAK5	LOC_Os01g70490	F:CTGATCTCAGAGGAGGACCTGCATATGACCGAGCCTCTGCACACA	pGBKT7

		R:GGCCGCTGCAGGTCGACGGATCCCGATCTCGTACGTCATTCCTACCCG	
OsJAMYB	LOC_Os11g45740	F:TACGACGTACCAGATTACGCTCATATGGAGATGGTGCTGCAGAGG	pGBKT7
		R:TGCAGCTCGAGCTCGATGGATCCCTTGCATCTTCCATATGTCCTCTAGACT	
OsMKK6	LOC_Os01g32660	F:CTGATCTCAGAGGAGGACCTGCATATGAGGGGGAAGAAGCCACAC	pGBKT7
		R:GGCCACTGCAGGTCGACGGATCCCTTACTOGGATATATTCATTGGAGGTTCAAG	
OsNAC77	LOC_Os12g03050	F:GCGACTAGTATGGTAGAGTCTACTACATCCCTTG	pGBKT7
		R:GCGCTCGAGCTGTCGTGATATGTAATGATCCCC	
OsNADPH	LOC_Os09g28570	F:CTGATCTCAGAGGAGGACCTGCATATGGAGCIGAAGCCCGGG	pGBKT7
		R:GGCCGCTGCAGGTCGACGGATCCCCTATAGCTTAGCAAGGGTCTGACTGTATGA	
OsPIIF	LOC_Os11g17790	F:CTGATCTCAGAGGAGGACCTGCATATGAGCAGCGACAAGTCGTCG	pGBKT7
		R:GGCCGCTGCAGGTCGACGGATCCCCTATCCGATCTTGGGGACCTG	
OsPP2C77	LOC_Os12g09640	F:GCGACTAGTATGCTCTGCGGCGTCGAG	pGBKT7
		R:GCGCTCGAGTACCOGCAAGAATGGTTGCAG	
OsTULP7	LOC_Os04g59130	F:CTGATCTCAGAGGAGGACCTGCATATGTCTTTCCGCAGCATAGTTCGT	pGBKT7
		R:GGCCGCTGCAGGTCGACGGATCCCTTATTCACATGCCAGCTTGGTGTC	
OsTULP11	LOC_Os07g47110	F:CTGATCTCAGAGGAGGACCTGCATATGTCTTTCAGGAGCGTGATCCAG	pGBKT7
		R:GGCCGCTGCAGGTCGACGGATCCCTCATTCGCACGCTATTTTGGTGTC	
OsUP	LOC_Os08g29160	F:CTGATCTCAGAGGAGGACCTGCATATGGCGCTCCATCTCACCACC	pGBKT7
		R:GGCCGCTGCAGGTCGACGGATCCCTCATTGCTTGGGAGACAATCTGATTGG	
OsCIPK17	LOC_Os05g04550	F:GCGACTAGTATGGTGAAGGGAGGGAGGGA	pSPYCE
		R:GCGCTCGAGCAAAGCGAACAGCGGC	
OsCIPK17	LOC_Os05g04550	F:GCGACTAGTATGGTGAAGGGAGGGAGGGA	pSPYNE
		R:GCGGGTACCCAAAGCGAACAGCGGC	
OsJAMYB	LOC_Os11g45740	F:GCGACTAGTATGGAGATGGTGCTGCAGAGG	pSPYNE
		R:GCGCTCGAGTTGCATCTTCCATATGTCCTCTAGACT	
OsNAC77	LOC_Os12g03050	F:GCGACTAGTATGGTGGAGTCTACTACATCCCTTGTG	pSPYNE
		R:GCGCTCGAGCTGTCGTGATATGTAATGATCCCC	
OsPP2C77	LOC_Os12g09640	F:GCGACTAGTATGCTCTGCGGCGTCGAG	pSPYCE
		R:GCGCTCGAGTACCGGCAAGAATGGTTGCAG	

Supplemental Table S2. GSEA scores from OsCBL8 overexpression lines based on KEGG database.

Name	Size	ES	NES	Nom p-val	FDR q-val
KEGG_DITERPENOID_BIOSYNTHESIS	20	0.67941135	1.7619492	0.002554278	0.08205975
KEGG_PROTEASOME	59	0.5541656	1.7025065	0.001091703	0.09123118
KEGG_PEROXISOME	74	0.5291559	1.6824982	0	0.076616526
KEGG_PHENYLALANINE	40	0.5556944	1.6188269	0.00116144	0.13585056
KEGG_PROTEIN_PROCESSING_IN_ENDOPLASMIC_RETICULUM	166	0.47049266	1.5788404	0	0.17810985
KEGG_ETHER_LIPID_METABOLISM	19	0.60639435	1.5491761	0.01858736	0.20417711
KEGG_PLANT-PATHOGEN_INTERACTION	76	0.49027136	1.5386949	0.003184713	0.19719848
KEGG_ENDOCYTOSIS	79	0.4799361	1.5382137	0.006362672	0.1730454
KEGG_FATTY_ACID_METABOLISM	41	0.52421266	1.5364838	0.012542759	0.15664856
KEGG_ABC_TRANSPORTERS	8	0.7290811	1.5105956	0.023255814	0.18215041
KEGG_SNARE_INTERACTIONS_IN_VESICULAR_TRANSPORT	35	0.5242221	1.4935495	0.030660378	0.19985627
KEGG_GLYCEROPHOSPHOLIPID_METABOLISM	55	0.4841889	1.4934614	0.01430143	0.1834433
KEGG_PENTOSE_PHOSPHATE_PATHWAY	44	0.5027312	1.4878788	0.022753129	0.17855978
KEGG_PHAGOSOME	72	0.4757875	1.4873545	0.006444683	0.16652119
KEGG_ALPHA-LINOLENIC_ACID_METABOLISM	30	0.5282627	1.4707478	0.03301887	0.1829122

Supplemental Table S3. GSEA scores from OsCIPK17 overexpression lines based on KEGG database.

Name	Size	ES	NES	Nom p-val	FDR q-val
KEGG_PROTEASOME	59	0.6443321	2.1190479	0	0
KEGG_N-GLYCAN_BIOSYNTHESIS	35	0.5766807	1.7446421	0.001148106	0.060925726
KEGG_SNARE_INTERACTIONS_IN_VESICULAR_TRANSPORT	33	0.56434804	1.6740121	0.002293578	0.10294486
KEGG_SPHINGOLIPID_METABOLISM	18	0.6392647	1.6730962	0.001251565	0.07868617
KEGG_GLYCEROPHOSPHOLIPID_METABOLISM	56	0.507327	1.6561309	0.001075269	0.08030397
KEGG_RNA_DEGRADATION	81	0.48030847	1.6312877	0.001055966	0.08718655
KEGG_GLUTATHIONE_METABOLISM	52	0.5031679	1.6128656	0.001091703	0.09500396
KEGG_CIRCADIAN_RHYTHM-PLANT	22	0.57959163	1.5844105	0.012077294	0.11669597
KEGG_ABC_TRANSPORTERS	8	0.70115864	1.5540072	0.02710027	0.14223565
KEGG_OXIDATIVE_PHOSPHORYLATION	94	0.45119783	1.5535579	0.004140787	0.12850812
KEGG_PHOSPHATIDYLINOSITOL_SIGNALING_SYSTEM	41	0.49944612	1.5328969	0.017563118	0.14523304
KEGG_PHAGOSOME	72	0.4538683	1.4888152	0.008474576	0.21212564
KEGG_UBIQUITIN_MEDIATED_PROTEOLYSIS	102	0.43443125	1.4881076	0.004149378	0.19743493
KEGG_ETHER_LIPID_METABOLISM	20	0.5442962	1.4816562	0.03789731	0.19484329
